# Interleukin 4 modulates microglia homeostasis and attenuates the early slowly progressive phase of amyotrophic lateral sclerosis

**DOI:** 10.1038/s41419-018-0288-4

**Published:** 2018-02-14

**Authors:** Chiara Rossi, Melania Cusimano, Martina Zambito, Annamaria Finardi, Alessia Capotondo, Jose Manuel Garcia-Manteiga, Giancarlo Comi, Roberto Furlan, Gianvito Martino, Luca Muzio

**Affiliations:** 1 Neuroimmunology Unit, Division of Neuroscience, Institute of Experimental Neurology (INSPE), San Raffaele Scientific Institute, 20132 Milan, Italy; 2 Centre for Translational Genomics and Bioinformatics, San Raffaele Scientific Institute, 20132 Milan, Italy; 3 Department of Neurology, Institute of Experimental Neurology (INSPE), Vita Salute San Raffaele University, 20132 Milan, Italy

## Abstract

Microglia activation is a commonly pathological hallmark of neurodegenerative diseases, such as amyotrophic lateral sclerosis (ALS), a devastating disorder characterized by a selective motor neurons degeneration. Whether such activation might represent a causal event rather than a secondary epiphenomenon remains elusive. Here, we show that CNS-delivery of IL-4—via a lentiviral-mediated gene therapy strategy—skews microglia to proliferate, inducing these cells to adopt the phenotype of slowly proliferating cells. Transcriptome analysis revealed that IL-4-treated microglia express a broad number of genes normally encoded by embryonic microglia. Since embryonic microglia sustain CNS development, we then hypothesized that turning adult microglia to acquire such phenotype via IL-4 might be an efficient in vivo strategy to sustain motor neuron survival in ALS. IL-4 gene therapy in SOD1^G93A^ mice resulted in a general amelioration of clinical outcomes during the early slowly progressive phase of the disease. However, such approach did not revert neurodegenerative processes occurring in the late and fast progressing phase of the disease.

## Introduction

Microglia are innate central nervous system (CNS) immune cells implicated in physiological and pathological processes^1^. They originate from yolk sack mesodermal precursors, seed the CNS during embryogenesis and account for 5–12% of mouse glial cells^2–4^. Microglia retain high mitotic activities during the development, while in the adult brain they are sustained by the local proliferation of slowly dividing progenitors^5–9^, and express distinct genetic pathways along the development^10^.

The initial response of microglia to the damage has been classified as M1 pro-inflammatory phenotype. Although anti-inflammatory cytokines such as Interleukin-4 (IL-4) and IL-13 skew cells to acquire the protective M2 alternative phenotype^11^. The identification of M1/M2 microglia markers is a recurrent issue in the recent research. With some limitations, markers like arginase-1 (Arg1), CD206, Resistin like alpha (Fizz1) and chitinase-like 3 (Ym1 in rodents) are currently used to identify M2 microglia^12–15^. However, in pathological conditions, the paradigmatic M1/M2 definition collapses in studies demonstrating that canonical markers of M1/M2 polarization phenotypes are co-expressed by single microglial cells^16–20^. Microglia show a surprising plasticity in response to environmental changes^21^, display regional heterogeneity in the CNS and respond to changes of gut microbiota, suggesting that they are versatile sensors of the extra- and the intra-CNS environment^10,22,23^.

IL-4 is a pleiotropic cytokine that regulates the brain homeostasis^24^ and supports oligodendrogenesis and neurogenesis^25–27^. Whilst IL-4 delivery in animal models of multiple sclerosis, brain ischemia and spinal cord injury is neuroprotective^28–33^, it has never been used in amyotrophic lateral sclerosis (ALS), a fatal disease featured by motor neurons (MNs) degeneration^34,35^. Microglia activation is a feature of ALS^36^, albeit it is not clear whether intrinsic mechanisms (protein aggregates) or extrinsic signals from dying MNs are driving such activation^28,29^. Similarly, the exact role of microglia in ALS pathophysiology is still ambiguous. For example, a reduction of reactive microglia has no influence on MNs survival^30^, although the blockade of CSF1R in SOD1^G93A^ (hereafter G93A) mice reduces “microgliosis”, slows disease progression and attenuates MN death^31^. Pharmacological and genetic inhibition of M1 polarization in microglia attenuates microglia-mediated MNs cell death and extends survival of mice^32,33,37^. However, the blockade of JAK2, neither increases motor performances nor extends life survival of mice^38^. The replacement of the G93A myeloid lineage with wild type (WT) microglia/macrophages or the reduction of infiltrating Ly6C^+^ cells increases mice survival^39–41^. In this complex scenario, microglia may participate in ALS pathogenesis as a double-edged sword, displaying negative effects, counterbalanced by neuroprotective responses.

## Results

### Interleukin-4 induces the expression of p-STAT6, Arg1 in a subset of spinal cold wild type microglia

**Fig. 1:**
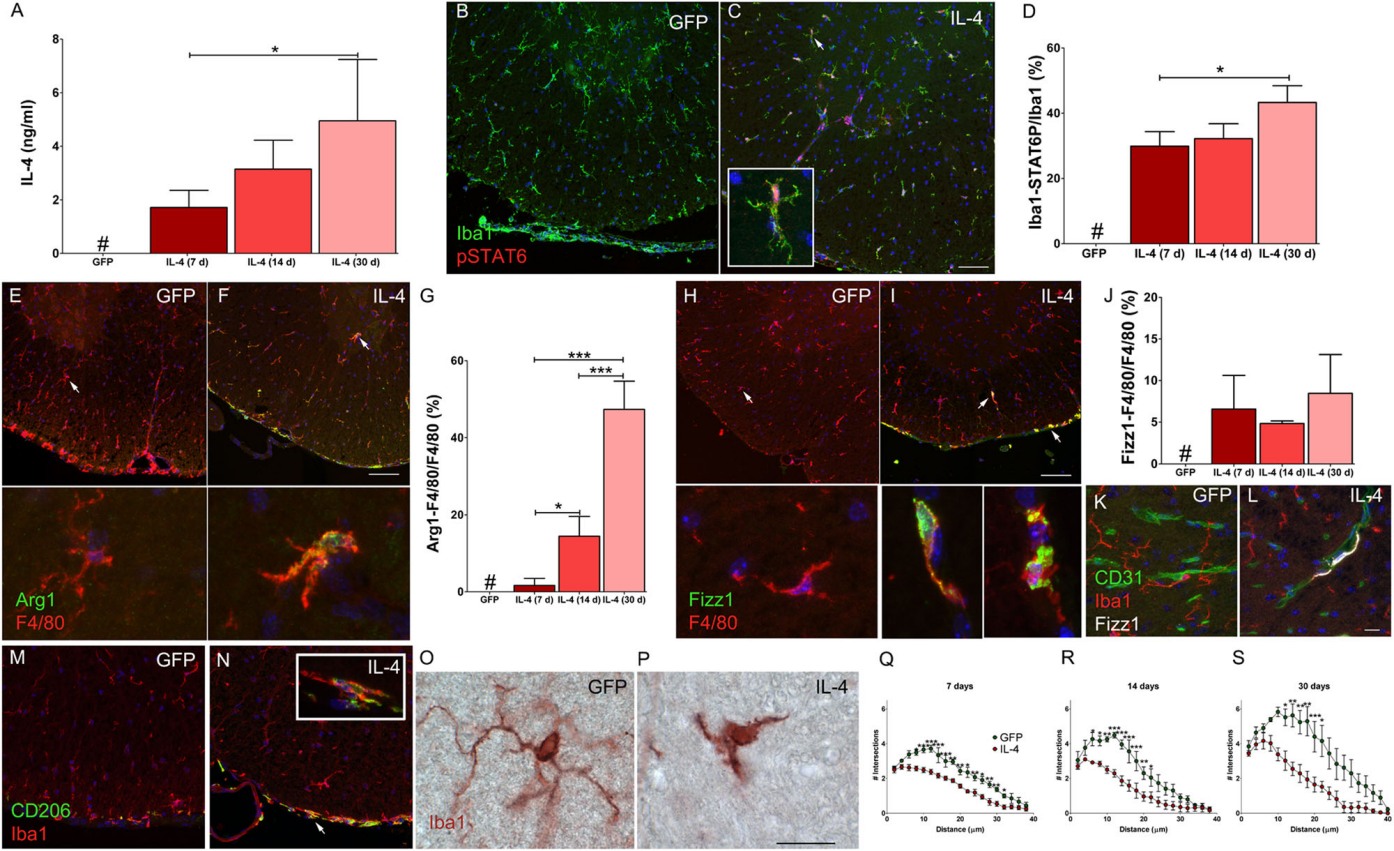
IL-4 enhances alternative activation of spinal cord microglia. **a** Production of IL-4 in the cerebrospinal fluid (CSF) of mice receiving LV and collected at day 7 (*n* = 4), 14 (*n* = 7), and 30 (*n* = 6). At each time point, IL-4 was undetectable in GFP-LV mice (*n* = 12). Results represent means ± S.D. **b** and **c** Immunofluorescence analysis on merged images for Iba1 and pSTAT6 from GFP-LV and IL-4-LV mice collected at day 14, arrow in C identifies a double positive cell shown in the inset. **d** Quantification (means ± S.D.) of ventral spinal cord Iba1/pSTAT6^+^ cells from GFP-LV (*n* = 9) and IL-4-LV mice at day 7, 14, and 30 (*n* = 3 for each time point). **e** and **f** Double immunofluorescences for Arg1 and F4/80 from GFP-LV and IL-4-LV mice collected at day 14. Arrows indicate cells that are shown at high magnification in bottom panels, no labeling were obtained in GFP-LV mice. **g** Percentages of double positive Arg1/ F4/80 cells (means ± S.D.) in GFP-LV (*n* = 9) and IL-4-LV mice at 7, 14, and 30 day (*n* = 3 for each time point). **h** and **i** Representative sections double labeled for Fizz1 and F4/80 from GFP-LV and IL-4-LV mice collected at day 14. Arrows indicate cells that are shown at high magnification in bottom panels, no labeling were obtained in GFP-LV mice. **j** Quantification of double positive Fizz1/ F4/80 cells (means ± S.D.) in GFP-LV (*n* = 9) and IL-4-LV mice at day 7, 14, and 30 (*n* = 3 for each time point). **k** and **l** Representative merged images for CD31, Iba1, and Fizz1 from GFP-LV and IL-4-LV mice. **l** and **m** Double immunofluorescences on merged images for CD206 and Iba1 from GFP-LV and IL-4-LV mice collected at day 14 (*n* = 3 for each group). **o**–**s** Representative images used for quantitative Sholl analysis (means ± S.E.M.) performed at day 7, 14, and 30 (*n* = 3 for each group at each time point). Data derive from Iba1 labeled sections from GFP-LV and IL-4-LV mice, numbers were obtained counting the number of branch tip intersections at increasing size of radius from the center of the soma with radial step size of 2 µm. One-way ANOVA followed by Bonferroni multiple comparisons post-test has been used to analyze data plotted in (**a**), (**d**), (**g**), and (**j**). Two-way ANOVA followed by Bonferroni multiple comparisons post-test has been used to analyze data in (**q**), (**r**), and (**s**). *p < 0.05; **p < 0.001; ***p < 0.001, # not detectable. Scale bar 50 µm in (**c**), (**f**), and (**i**): 10 µm in (**l**) and (**p**)

We injected IL-4 or GFP lentiviral vector (LV) in the fourth ventricle of P30 naïve mice^42^. Spinal cords (SC) were collected 7, 14 and 30 days after LV delivery. IL-4-LV mice looked healthy, not showing any behavioral phenotype^43^ nor displaying abnormalities of the CNS gross anatomy (not shown). IL-4 was observed in the cerebrospinal fluid (CSF) of IL-4-LV mice, while it was negligible in GFP-LV mice (Fig. 1a). We assayed SC sections for phosphorylated signal transducers and activators of transcription (STAT) 6^44^. Phospho-STAT6^+^Iba1^+^ cells were only detected in IL-4-LV mice and percentages of double positive cells increased up to 43% at day 30 (Figs. 1b-d). We next scored Arg1 and Fizz1 in F4/80^+^ microglia^45–49^. IL-4 induced Arg1 in few parenchymal F4/80^+^ cells at day 7, while their percentages increased up to 47% at day 30 (Figs. 1e-g). Few parenchymal F4/80^+^ cells co-expressed Fizz1 in IL-4-LV mice, while the vast majority of meningeal macrophages expressed Fizz1. Percentages of parenchymal F4/80^+^Fizz1^+^ cells were pretty stable along the time (Figs. 1h-j). The vast majority of these cells were in close proximity to CD31^+^ endothelial cells (Figs. 1k, l) and, on the basis of their distinct cell morphology, they could be referred as peri-vascular macrophages^50^. CD206 was confined in meningeal macrophages, while parenchymal microglia were always negative (Figs. 1m, n)^42^. Arg1, Fizz1 and CD206 levels were negligible in GFP-LV mice (Figs. 1e, h, k and m).

We explored Arg1, Fizz1 and CD206 in primary microglia cultures. Cells received increasing amount of recombinant IL-4 and then *Iba1*, *CD206*, *Arg1*, and *Fizz1* mRNA levels were assayed by real time PCR. The expression levels of *Iba1* did not change in response to the IL-4 treatment, while *CD206*, *Arg1*, and *Fizz1* mRNA levels were greatly increased by IL-4, (Supplementary figure 1A, B). Parallel cultures received IL-4 (20 ng/ml) and were labeled for Arg1, Fizz1, and CD206. Very few IB4^+^Arg1^+^ or IB4^+^Fizz1^+^ cells were detected in untreated cultures (Supplementary figure 1C and F), while around 30% of untreated IB4^+^ cells were CD206^+^ (Supplementary figure 1I and K). IL-4 slightly, but significantly, increased percentages of double positive cells, mirroring results that we obtained in vivo (Supplementary figure 1G, H, J and K). Microglia/macrophages activation/polarization is often accompanied by changes of cell morphology^51^. We measured branching of Iba1^+^ cells through a Sholl analysis^52^. Microglia of GFP-LV mice displayed a small cell body and thin and highly ramified processes that project in the parenchyma (Fig. 1o)^53^. In contrast, microglia of IL-4-LV mice shortened processes and limited the number of segments, showing a cell morphology that was reminiscent of embryonic/early post-natal microglia (Figs. 1p-s)^54^.

Because IL-4 could directly or indirectly attract some cells from the hematopoietic system to infiltrate the CNS we asked whether blood borne monocytes invade the CNS. We transplanted bone marrow cells (BMT) from GFP donors^55^ in mice receiving treosulfan (TREO) as conditioning regimen. TREO is unable to cross the Blood Brain Barrier, thus it does not allow microglia depletion and reconstitution following BMT^56^. Two months after BMT, percentages of GFP^+^CD45^+^CD11b^+^ blood cells ranged from 98.1 ± 1.7%. TREO-BMT mice received IL-4 and GFP LVs and SCs were scored for Iba1^+^GFP^+^ cells. We did not observe any Iba1^+^GFP^+^ cells in the CNS parenchyma of IL-4-LV and GFP-LV mice, although some double positive cells were detected in meninges of both groups of mice (Supplementary figure 2A-D).

### Interleukin-4 stimulates microglia cell proliferation

**Fig. 2:**
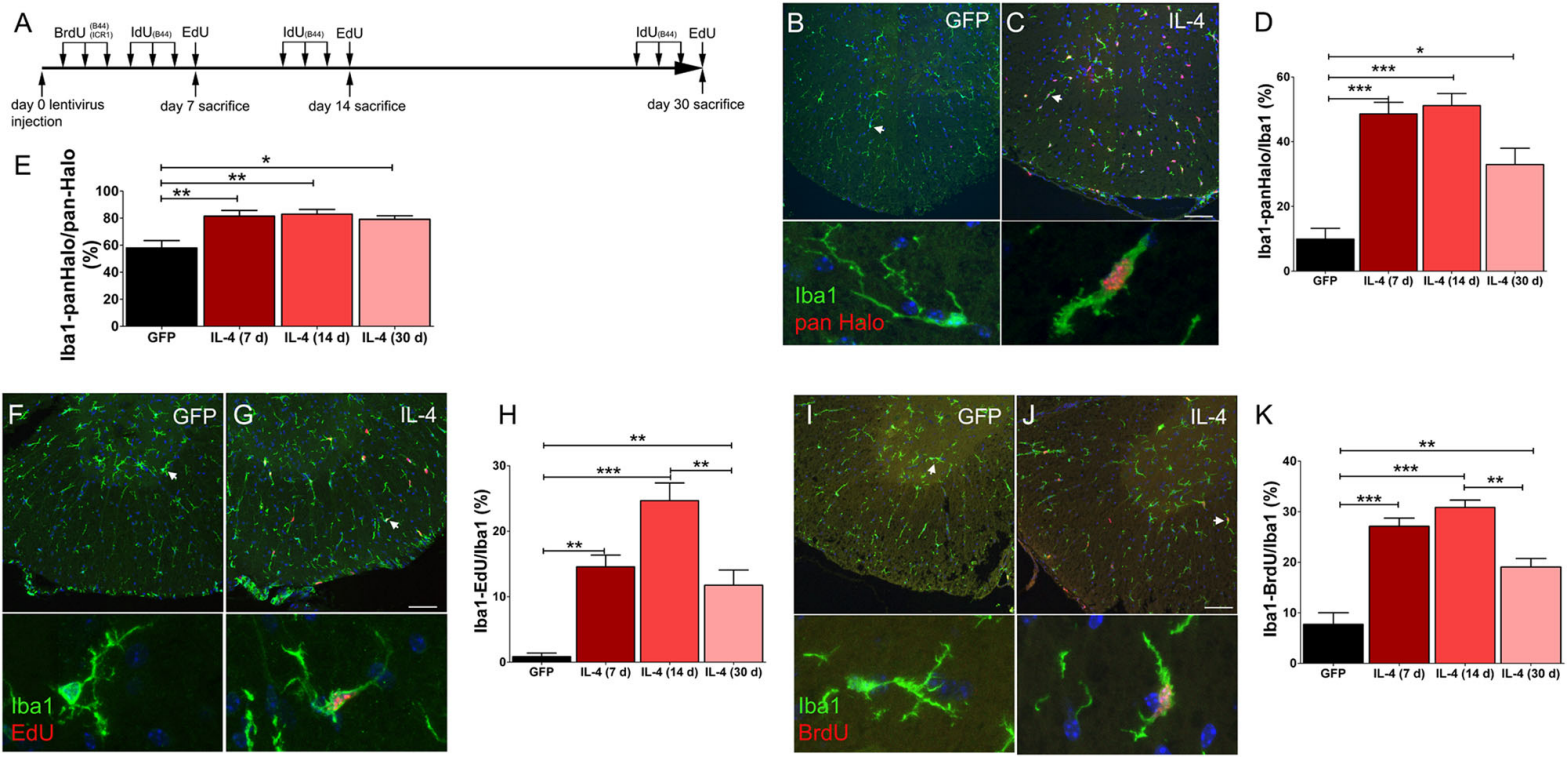
IL-4 enhances the uptake of S-phase tracers in spinal cord microglia. **a** Schematic representation of the S-phase tracers labeling paradigm. **b** and **c** Merged images of lumbar sections from GFP-LV and IL-4-LV mice collected at day 14 and double labeled for Iba1 and with the anti-pan Halogen antibody. Arrows indicate cells that are shown at high magnification in bottom panels. **d** Percentages of Iba1/pan Halogen positive cells among Iba1 cells. Data are represented as mean ± S.E.M. of GFP-LV mice (*n* = 4), IL-4-LV mice at day 7 (*n* = 6), IL-4-LV mice at day 14 (*n* = 5) and IL-4-LV mice at day 30 (*n* = 3). **e** Quantification of double positive Iba1/pan Halo cells among pan-Halogen^+^ cells. Data are represented as mean ± S.E.M. of GFP-LV mice (*n* = 4), IL-4-LV mice at day 7 (*n* = 6), IL-4-LV mice at day 14 (*n* = 5) and IL-4-LV mice at day 30 (*n* = 3). **f** and **g** Merged images showing immunofluorescence for Iba1 and EdU in GFP-LV and IL-4-LV mice at day 14. Arrows indicate cells that are shown at high magnification in bottom panels. **h** Percentages of double positive Iba1/EdU cells. Data are represented as mean ± S.E.M. of GFP-LV mice (*n* = 9), IL-4-LV mice at day 7 (*n* = 4), IL-4-LV mice at day 14 (*n* = 4) and IL-4-LV mice at day 30 (*n* = 4). **i** and **j** Representative merged images of lumbar spinal cord sections double labeled for Iba1 and BrdU. Arrows indicate cells that are shown at high magnification in bottom panels. **k** Quantification of double positive Iba1/BrdU cells. Data are represented as mean ± S.E.M. of GFP-LV mice (*n* = 9), IL-4-LV mice at day 7 (*n* = 6), IL-4-LV mice at day 14 (*n* = 5) and IL-4-LV mice at day 30 (*n* = 3). One-way ANOVA followed by Bonferroni’s multiple Comparison test has been used to analyze plots in panels (**d**), (**e**), (**h**), and (**k**). *p < 0.05; **p < 0.001; ***p < 0.001. Scale bar 50 µm in (**c**), (**g**), and (**j**); 10 µM in (**m**) and 30 µM in (**p**)

**Fig. 3:**
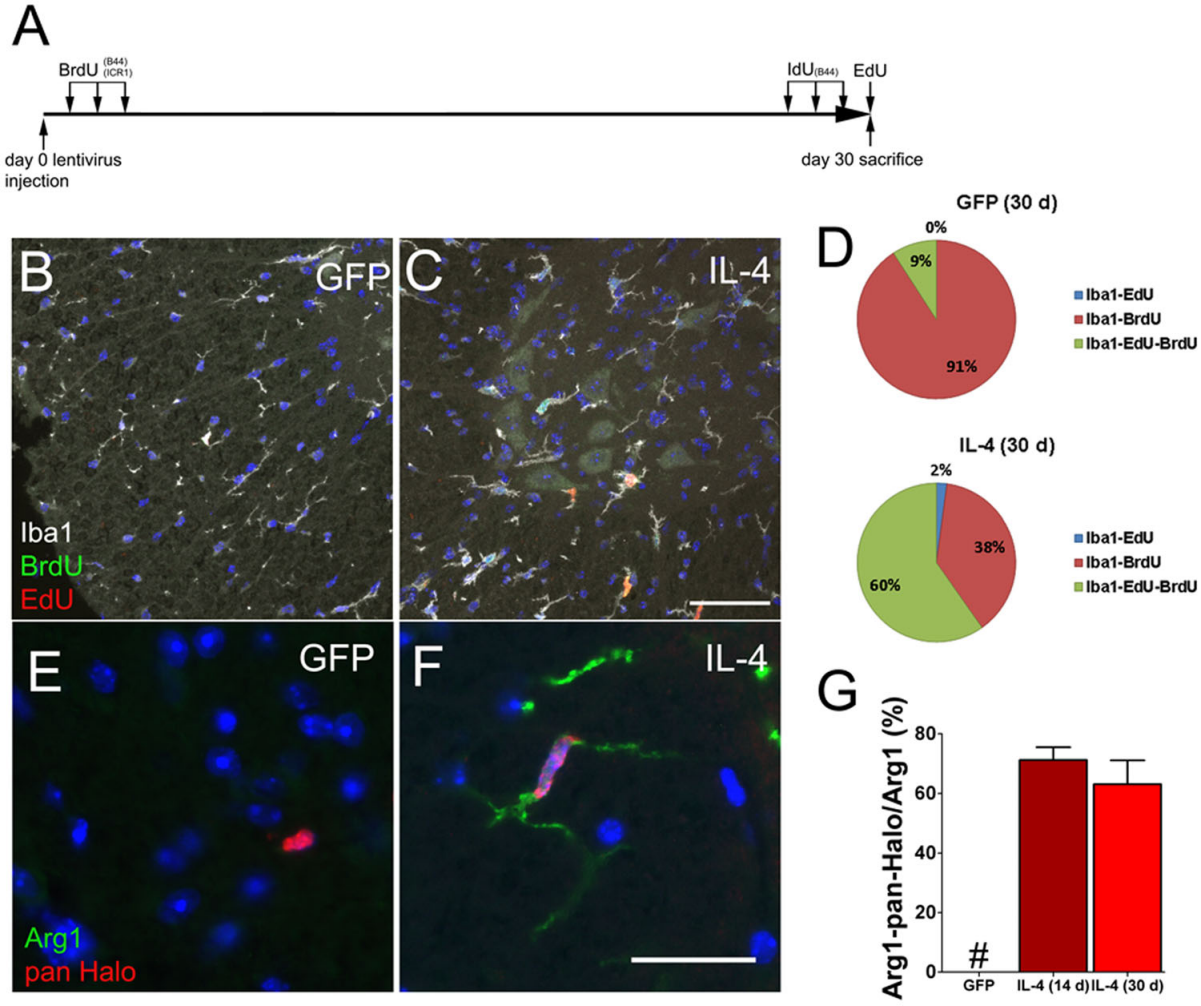
IL-4 increases S-phase tracers’ incorporation in Arg1 expressing cells. **a** Schematic representation of the labeling paradigm. **b** and **c** Representative triple immunofluorescence for Iba1, BrdU and EdU from GFP-LV and IL-4-LV mice collected at day 30. **d** Quantifications of Iba1/ BrdU/ EdU positive cells on GFP-LV mice (*n* = 4) and IL-4-LV mice (*n* = 6), (GFP: Iba-EdU 0%; Iba-BrdU 91 ± 5%; and Iba-EdU-BrdU 9 ± 5%); (IL-4: Iba-EdU 2 ± 1.6%; Iba1-BrdU 38 ± 25%; and Iba1-EdU-BrdU 60 ± 26%). **e** and **f** Double labeled sections for Arg1 and an anti- pan Halogen antibody from GFP-LV and IL-4-LV mice. **g** Percentages (mean ± S.D.) of double positive Iba1/Arg1 cells in IL-4-LV mice (14d *n* = 5, 30d *n* = 3). GFP-LV mice (*n* = 5) did not show any double positive cells. One-way ANOVA followed by Bonferroni’s multiple Comparison test has been used to analyze data in (**g**). Scale bar 50 µm in (**c**) and 10 µm in (**f**). # not detectable

At steady state the microglial self-renewal is a stochastic process^57^ maintained by a slowly proliferating and long lived cells^9,58^. IL-4 modulates macrophages cell renewal^59^ and increased microglia cell proliferation in vitro^60^. We assayed microglia cell proliferation using different S-phase tracers (see the experimental paradigm in Fig. 2a). Using an anti-pan-Halogen antibody we scored cells incorporating either BrdU or IdU tracers (Figs. 2b, c). Percentages of pan-Halogen^+^Iba1^+^ were less than 10% in GFP-LV mice and did not increase along the time. Thus, we averaged values from GFP-LV mice collected at each time point and we used their mean value as reference (Figs. 2b, d). In IL-4-LV mice, percentages of pan-Halogen^+^Iba1^+^ cells increased at day 7, reached the highest value at day 14, while slightly decreased at day 30 (Figs. 2d, e). The use of the pan-Halogen antibody does not allow to discriminate between cells that did BrdU uptake soon after the LV delivery and cells that incorporated IdU tracer at the end of the treatment. Thus, we exploited the EdU to define percentages of proliferating microglia at each time point^61^. Few Iba1^+^EdU^+^ cells were detected in GFP-LV mice (Fig. 2f)^6,9^. IL-4 increased percentages of double positive cells that peaked at day 14, while declined at day 30 (Figs. 2g, h). BrdU injections label microglia soon after the LV delivery (Fig. 2a). We would expect that cells displaying a slow cell cycle or escaping the cell cycle do not dilute the BrdU-label. On the other hand, fast proliferating cells dilute S-phase tracer contents below the detectable threshold. In IL-4-LV mice as compared to controls, high numbers of Iba1^+^ cells retained BrdU labeling and percentages of Iba1^+^BrdU^+^ cells peaked at day 14 (Figs. 2i-k). We next scored for S phase reentry of microglia at day 30, by triple labeling sections for Iba1, EdU, and BrdU (Fig. 3a). Percentages of triple positive cells were markedly increased in IL-4-LV mice (Figs. 3b-d), suggesting that IL-4 fosters microglia to stay mitotically active with prolonged cell cycle reentry times. Seeking for a correlation between cell proliferation and microglia cell density, we scored SC sections for Iba1. Microglia cell density did not increase at day 7, while a mild, but significant, increase was observed at day 30 (Supplementary figure 3A-C). However, IL-4 did not shorten the length of cell cycle of microglia, as shown by scoring numbers of Iba1^+^ cells expressing the M-phase marker phospho-Histone 3 (pH3), (Supplementary figure 3D-F). Because IL-4 can increase microglia cell death in vitro^62^, we labeled sections for Iba1 and cleaved Caspase 3 (CC3). However, we did not observe any significant change of cell death rates in IL-4-LV mice as compared to GFP-LV controls (Supplementary Figure 4B-D). We next labeled sections for the pan-Halogen and Arg1 to assess whether any correlation between cell proliferation and the expression of Arg1 may exist. GFP-LV mice did not express Arg1 (Fig. 1e), and the few pan-Halogen^+^ cells that we scored were negative for Arg1 (Fig. 3e). In contrast, 71 and 63% of Arg1^+^ cells were pan-Halogen^+^ in IL-4-LV mice, at day 14 and day 30, respectively (Figs. 3f, g).

### Interleukin-4 fosters microglia to express a developing gene signature

**Fig. 4:**
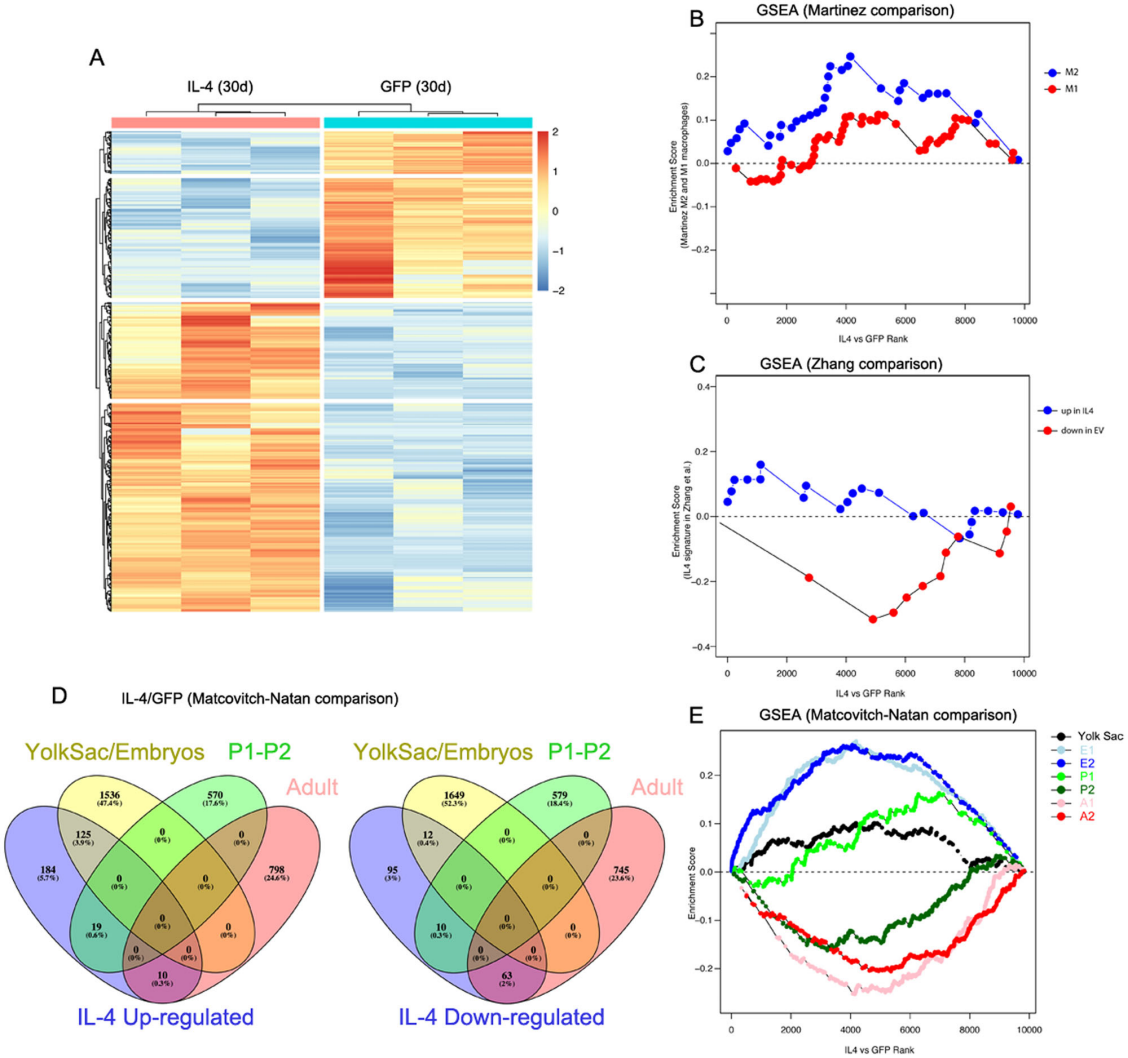
Gene expression patters of IL-4 treated microglia. **a** Heatmap of genes differentially expressed in microglia of IL-4-LV mice compared with microglia of GFP-LV mice (*n* = 3 for each group) by RNA-seq, ranked by fold change (FDR < 0.05). **b** GSEA showing the enrichment for data set provided by Martinez et al. ^63^, showing M1 and M2 signatures established on human and mouse macrophages. Normalized Enriched Score (NES) for the M1 = 0.95, *p* = 0.5; NES for M2 = 1.65, *p* = 0.04. **c** GSEA showing the enrichment for data set provided by Zhang et al. ^65^, in which the IL-4 gene therapy has been applied to mammary carcinoma cells, NES empty vector (EV) treated cells = −1.63, *p* = 0.03; NES for IL-4 treated cells = 0.89, *p* = 0.6. **d** Venn diagrams showing unique and intersecting genes up-regulated (left panel) and downregulated (right panel) in IL-4-treated microglia compared with published data set showing microglia enriched genes in yolk sac/embryonic, post-natal (P1 and P2) and adult microglia. **e** GSEA showing the enrichment for genes reported by signatures by Matcovitch-Natan et al. ^10^ NES for Yolk Sac = 1.99, *p* = 0.008; NES for embryonic E1 microglia = 7.79, *p* < 0.001; NES for embryonic E2 microglia = 6.23, *p* < 0.001; NES for postnatal P1 microglia = 2.64, *p* < 0.001; NES for postnatal P2 microglia = −3.36, *p* < 0.001; NES for adult A1 microglia = −5.53, *p* < 0.001 and NES for adult A2 microglia = −4.49, *p* < 0.001

We next performed an RNA-seq analysis of CD45^low^CD11b^+^ cells isolated from IL-4-LV and GFP-LV mice. Hierarchical clustering of genes with differential expression segregated IL-4 microglia (Fig. 4a, Supplementary Table I). Using the package DESeq2 and a FDR < 0.05, we identified 518 genes that were differentially regulated in IL-4-LV mice. Among them, 338 were upregulated, while 180 were down-regulated in IL-4-LV microglia. As expected, *Arg1* and *Fizz1* mRNAs were significantly enriched in IL-4-LV microglia. Among the most up-regulated RNAs we identified genes linked to the cell cycle, such as *Cdkn2a*, *Cdc20*, *Cdk1, AspM, Ccna2*, and *Mki67* (Supplementary Table I).

IL-4 induces a specific gene signature in human and mice macrophages as shown by Martinez and coworkers^63^. We used this database to perform a Gene Set Enrichment Analysis (GSEA)^64^. We observed a poor enrichment for genes expressed by M1 and M2 polarized macrophages with a Normalized Enriched Score (NES) = 1.65, *p* = 0.04 and NES = 0.95, *p* = 0.5 for M2 and M1 gene lists, respectively (Fig. 4b). IL-4 reduces growth and metastasis of breast cancer cells acting on tumor associated myeloid cells^65^. Using a NanoString approach, authors of this study showed a specific immune signature of myeloid cells^65^. However, NES values also indicated a poor correlation with this database (NES = −1.63, *p* = 0.03 for genes differentially regulated in cells receiving the empty vector; NES = 0.89, *p* = 0.6 for genes modulated by IL-4), (Fig. 4c). Microglia are featured by a specific genetic program that evolves from the early development to the adulthood^10^. Venn diagram showed that among up-regulated genes in IL-4-LV microglia, we found that 125 genes were in common with yolk sac/embryonic microglia, 19 with P1-P2 microglia, while only 10 were expressed by adult microglia. On the other hand, among down-regulated genes in IL-4-LV microglia, 63 genes were in common with adult microglia, while only 12 and 10 genes were in common with yolk sac and post-natal microglia, respectively (Fig. 4d). GSEA revealed a good correlation with the dataset published by Matcovitch-Natan^10^, with higher values of NES for genes expressed by embryonic (E) microglia and upregulated in IL-4-LV microglia (NES E1 = 7.79, *p* < 0.001; NES E2 = 6.23, *p* < 0.001). When we did GSEA of genes modulated in IL-4-LV microglia with gene lists representing adult (A) microglia, we observed that they were substantially depleted (NES A1 = −5.53, *p* < 0.001; NES A2 = −4.49, *p* < 0.001).

### Interleukin-4 induces M2 genes in G93A microglia

**Fig. 5:**
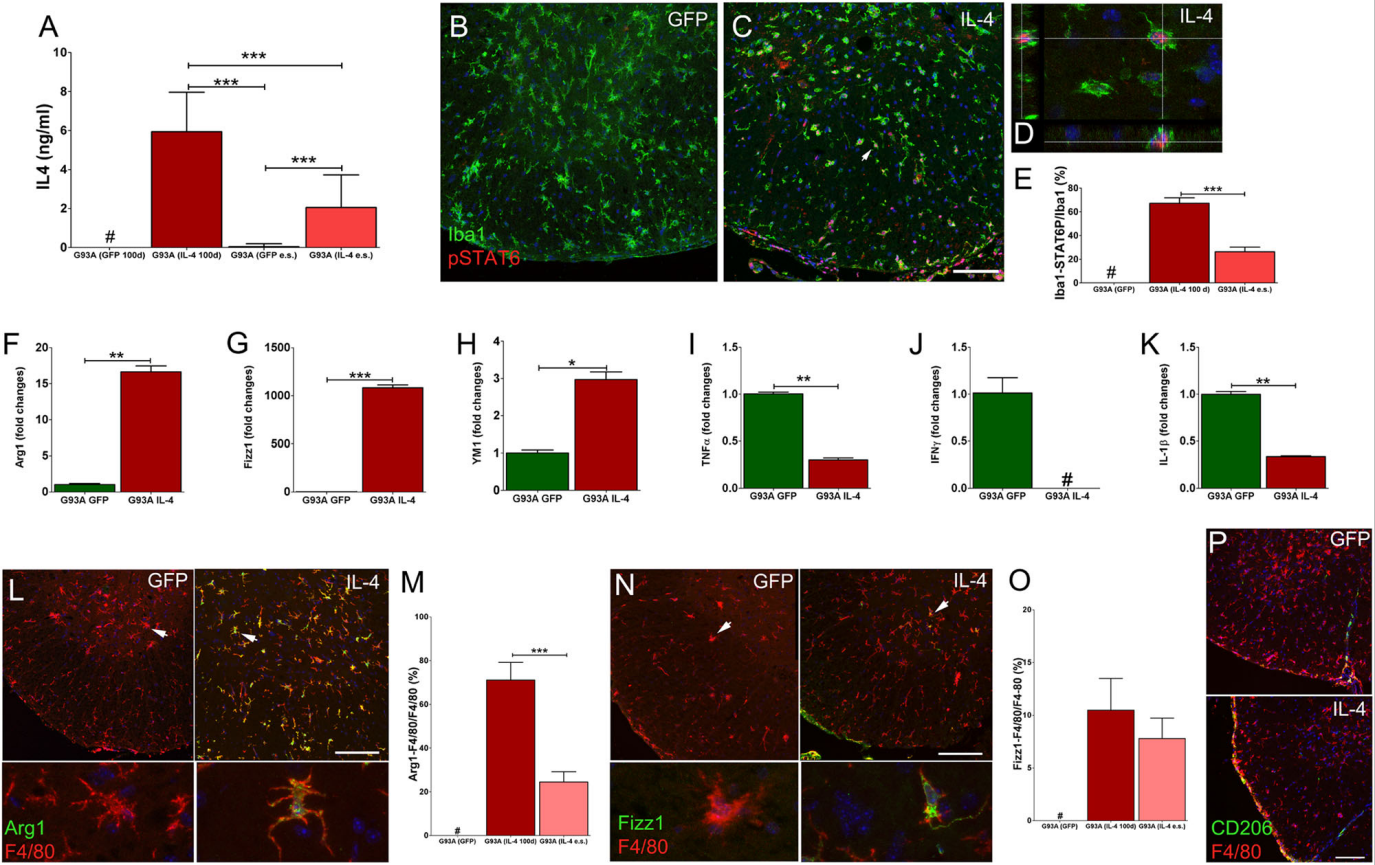
IL-4 enhances alternative activation of G93A spinal cord microglia. **a** CSF levels of IL-4 (mean ± S.D.) in G93A-GFP-LV mice and G93A-IL-4-LV mice. CSF was retrieved at day 100 (*n* = 4 per group) and at end stage (GFP *n* = 13, IL-4 *n* = 11). **b**, **c**, and **d** Double labeling for Iba1 and pSTAT6 on merged images from G93A-GFP-LV (**b**) or G93A-IL-4-mice (**c**, **d**) collected at end stage (end stage). Arrow in C indicates a single cell that is shown at high magnification in **d**. **e** Percentages (mean ± S.D.) of Iba1/pSTAT6^+^ cells at day 100 and at end stage (*n* = 3 for each group). **f**–**k** Real time PCR analysis for genes encoding bona fide M2 markers (**f**–**h**) and pro inflammatory markers (**i**–**k**) in purified microglia. Values indicate the mean fold change ± S.E.M. from two independent experiments (*n* = 6 for each group). **l** Double immunofluorescence for Arg1 and F4/80 on stitching images from G93A-GFP-LV and G93A-IL-4-LV mice (day 100). Arrows indicate cells that are shown at high magnification in bottom panels. **m** Quantification (mean ± S.D.) of double positive Arg1/F4/80 cells obtained from 100 days and end stage mice (*n* = 3 for each group). **n** Representative sections labeled for Fizz1 and F4/80 from G93A-GFP-LV mice and G93A-IL-4-LV mice. Arrows indicate cells that are shown at high magnification in bottom panels. **o** Percentages (mean ± S.D.) of double positive Fizz1/ F4/80 cells from 100 days and end stage mice (*n* = 3 for each group). **p** Representative double immunofluorescence for CD206 and F4/80 on stitching images from G93A-GFP-LV mice and G93A-IL-4-LV mice sampled at end stage (*n* = 3 for each group). One-way ANOVA followed by Bonferroni multiple comparisons post-test has been used to analyze data plotted in A. Unpaired *t* test has been used to analyze data plotted on panels (**e-k**) and (**m**). *p < 0.05; **p < 0.01; ***p < 0.001, # not detectable. Scale bar 50 µm

We next addressed the impact of IL-4-LV on neurodegeneration in mice carrying the G93A allele^66^. ALS mice received IL-4 LV at day 70, a time point in which mice are asymptomatic and almost all Iba1^+^cells display a cell morphology that reflects their non-activated state (Supplementary Figure 5A-D)^30,67^. Similarly, numbers and cell morphology of choline acetyltransferase^+^ (ChAt) MNs were unaffected (Supplementary Figure 5E, F). Mice were sacrificed 30 days after LV delivery (day 100) or at end stage (17–21 weeks). IL-4 levels were undetectable in the CSF of G93A-GFP-LV mice, while expressed in G93A-IL-4-LV mice (Fig. 5a). At day 100, G93A-GFP-LV mice did not express p-STAT6 (Fig. 5b), while 60% of Iba1^+^ cells were pSTAT6^+^ in G93A-IL-4-LV mice (Figs. 5c-e). However, percentages of Iba1^+^pSTAT6^+^ cells significantly declined in end stage mice, mirroring the reduction of IL-4 levels (Fig. 5a,e). We assayed the expression levels of *Arg1*, *Fizz1*, *Ym1 TNFα*, *IFNγ*, and *IL-1β* mRNAs on CD11b^+^ cells (Supplementary Figure 6A, B)^68^. IL-4 increased the expression levels of *Arg1*, *Fizz1*, and *Ym1* in microglia (Figs. 5f-h), and concurrently decreased the expression of pro-inflammatory cytokines (Figs. 5i-k). We next labeled sections for Arg1, Fizz1 and CD206 in G93A-GFP-LV and G93A-IL-4-LV mice. Such markers were negligible in G93A-GFP-LV mice (Figs. 5l, n and p), while more than 70% of microglia expressed Arg1 in G93A-IL-4-LV mice (Figs. 5l, m). Fizz1 was expressed by a small cohort of microglia and percentages of Fizz1^+^F4/80^+^ cells did not increase along the time (Figs. 5n,o). CD206 was undetectable in parenchymal microglia of both G93A-GFP-LV and G93A-IL-4-LV mice while high levels were observed in meningeal macrophages of G93A-IL-4-LV mice (Fig. 5p).

### IL-4-increases microglia cell proliferation in G93A mice

**Fig. 6:**
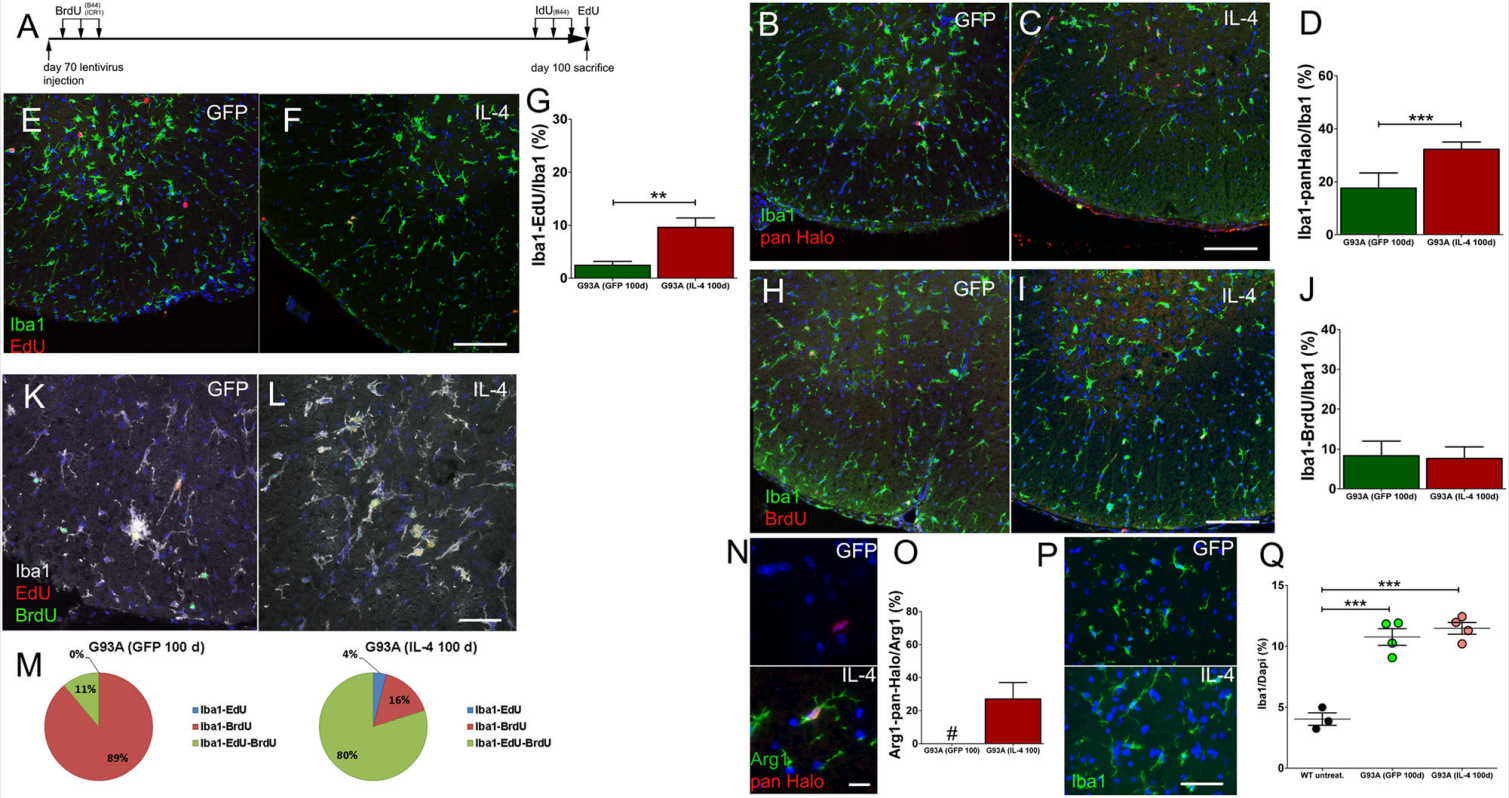
IL-4 triggers microglia cell proliferation in G93A mice. **a** Schematic representation of the S-phase tracers labeling paradigm. **b** and **c** Merged images of lumbar sections from G93A-GFP-LV and G93A-IL-4-LV mice labeled for Iba1 and with the anti-pan Halogen antibody. **d** Percentages of double positive Iba1/pan Halogen cells (*n* = 4 for each group). **e** and **f** Double labeling on stitching images for Iba1 and EdU of lumbar sections from G93A-GFP-LV mice and G93A-IL-4-LV mice. **g** Quantification of double positive Iba1/EdU cells (*n* = 4 for each group). **h** and **i** Merged images of lumbar spinal cord sections double labeled for Iba1 and BrdU. **j** Percentages of double positive Iba1/BrdU cells (*n* = 4 for each group). **k** and **l** Triple immunofluorescence for Iba1, BrdU, and EdU on G93A-GFP-LV and G93A-IL-4-LV mice. **m** Quantifications of Iba1/BrdU/ EdU positive cells (*n* = 4 for each group); (GFP: Iba-EdU 0%; Iba-BrdU 89 ± 19%; and Iba-EdU-BrdU 11 ± 19%); (IL-4: Iba-EdU 4 ± 4%; Iba1-BrdU 16 ± 26%; and Iba1-EdU-BrdU 90 ± 29%). **n** Representative sections from G93A-GFP-LV mice and G93A-IL-4-LV mice labeled for Arg1 and anti-pan Halogen antibody. **o** Percentages (mean ± S.D.) of double positive Arg1/Pan Halogen cells in G93A-IL-4-LV mice, G93A-GFP-LV mice did not show any double positive cells (*n* = 4 for each group). **p** and **q** Immunofluorescence for Iba1 and cell densities counts (each dot represents a single mouse). Unpaired *t* test has been used to analyze data plotted on panels (**d**), (**g**), and (**j**). One-way ANOVA followed by Bonferroni’s multiple Comparison test has been used to analyze data in panel (**q**), **p < 0.001; ***p < 0.001. Scale bar 50 µm in (**c**), (**f**), (**i**), and (**l**); 10 µM in (**n**) and 30 µM in (**p**)

We next used S-phase tracers to study microglia proliferation (Fig. 6a). We initially scored proliferating microglia using the anti-pan-Halogen antibody. Because a mild “microgliosis” is featuring the spinal cord of 12–16 weeks G93A mice^30,31^, we found that percentages of proliferating microglia were doubled in G93A-GFP-LV mice when compared with GFP-LV mice (Figs. 6b-d and Fig. 2d). IL-4 further increased percentages of proliferating cells in the G93A background (Figs. 6c, d). Numbers of EdU^+^ microglia were significantly increased by IL-4 (Figs. 6e-g). However, both G93A-IL-4-LV and G93A-GFP-LV mice displayed similar percentages of BrdU^+^ microglia (Figs. 6h-j). We triple labeled sections for Iba1, BrdU and EdU to identify long lasting proliferating cells. Low percentages of triple positive cells and high percentages of Iba1^+^BrdU^+^ cells were observed in G93A-GFP-LV mice. IL-4 markedly increased percentages of both triple positive cells and Iba1^+^EdU^+^ cells (Figs. 6k-m). However, only 28% of cells incorporating S-phase tracers were also positive for Arg1 in G93A-IL-4-LV mice (Figs. 6n, o). The Iba1^+^ cell density was higher in G93A mice receiving GFP- and IL-4-LV than in WT mice^30^, although IL-4 did not increase this number (Figs. 6p, q). Finally, Iba1^+^CC3^+^ cells were slightly, but not significantly, increased by IL-4 (Supplementary figure 4E).

### Interleukin 4 improves locomotion, delays the disease onset but does not extend the life span of G93A mice

**Fig. 7:**
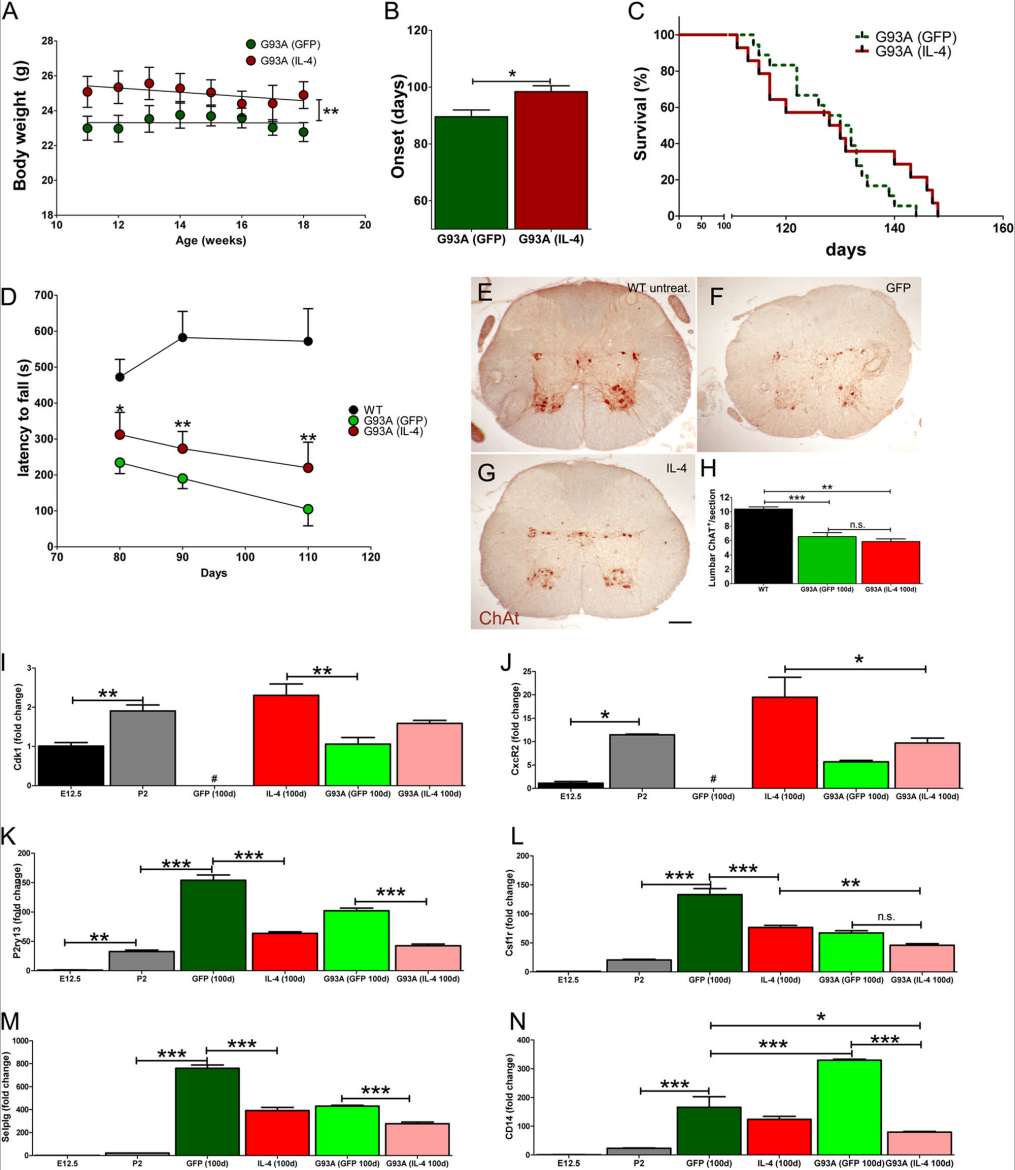
Effects of IL-4 gene therapy on the phenotype, disease onset and life span of SOD1^G93A^ mice. **a** G93A-IL-4-LV mice displayed higher body weight than G93A-GFP-LV mice. G93A-IL-4-LV maintained an overall significant higher weight until the day of the sacrifice (each symbol represents the mean ± S.E.M., *n* = 9 for each group). Linear regression analysis shows that the elevation between the two curves is significant, *p* < 0.01. **b** Clinical onset of the disease in G93A-GFP-LV mice (*n* = 18) and G93A-IL-4-LV mice (*n* = 10). **c** Kaplan-Meyer of survival of G93A-GFP-LV mice (*n* = 18, median survival 131 days) and G93A-IL-4-LV mice (*n* = 14, median survival 129 days). **d** Rota-rod tests detected increased time remained on rota-rod in G93A-IL-4-LV mice. Untreated WT mice (*n* = 5), G93A-GFP-LV mice (*n* = 5) and G93A-IL-4-LV mice (*n* = 4). Mice were placed on an accelerated rod with speed from 4 to 40 rpm over 15 min. The latency to fall off the rota-rod was recorded at day 80, 90, and 110, and means ± S.E.M. plotted in the histogram. **e**–**g** Immunohistochemistry for ChAt in untreated WT mice, G93A-GFP-LV mice and G93A-IL-4-LV mice at day 100. **h** Quantifications of ChAt^+^ neurons scored in the ventral horn of the lumbar spinal cord at day 100 (*n* = 3 for each group). **i**–**n** Real time PCR for *Cdk1*, *CxcR2*, *P2ry13*, *Csf1R*, *Selplg*, and *CD14* on microglia from E12.5 (*n* = 3), P2 (*n* = 3), GFP-LV (*n* = 4), IL-4-LV (*n* = 4), G93A-GFP-LV (*n* = 5) and G93A-IL-4-LV (*n* = 6). Data are represented as mean ± S.E.M. Data in (**b**) has been analyzed by Mann–Whitney test, while Log-rank (Mantel–Cox) Test was used to analyze Kaplan–Meyer data of panel (**c**). Data of panels (**d**) have been analyzed by Kruskal–Wallis test followed by Dunn’s Multiple Comparison Test. Unpaired *t* test has been used to analyze data plotted on panel (**h**). One way ANOVA followed by Tukey’s Multiple Comparison Test has been used to analyze data in panels (**i–n**). *p < 0.05; **p < 0.01; ***p < 0.001. Scale bar 100 µm

Starting from day 74, G93A-GFP-LV and G93A-IL-4-LV mice were body weighted for the consecutive 9 weeks. G93A-IL-4-LV mice showed a significant higher body mass than G93A-GFP-LV mice (Fig. 7a). IL-4 significantly delayed the disease onset of mice (Fig. 7b), but unfortunately, did not prolong their life span (Fig. 7c). We next monitored motor performances using the accelerated rota-rod test. IL-4 slowed the progression of motor impairment as witnessed by the increased latency to fall that we observed in G93A-IL-4-LV mice (Fig. 7d). Immunohistochemical evaluation of ChAt^+^ MNs at day 100 revealed similar numbers of cells in G93A-GFP-LV and G93A-IL-4-LV mice (Fig. 7e-h). We sorted CD45^low^CD11b^+^ cells from G93A-GFP-LV and G93A-IL-4-LV mice at day 100 to investigate genes that were modulated by IL-4 (Fig. 4a)^10^. We also established, as control, microglia sorted from embryonic (E12.5) and post-natal (P2) brains. *Cdk1* and *CxcR2* were enriched in E12.5 and P2 microglia, while negligible in microglia from adult GFP-LV mice^10^ and they were increased both in IL-4-LV and G93A-GFP-LV mice. Differences observed in microglia of G93A-GFP-LV and G93A-IL-4-LV mice were a trend, not reaching the statistical significance (Figs. 7i, j). *P2ry13* is a G protein-coupled nucleotide receptors involved in microglia activation^69^. It was highly expressed by adult GFP-LV microglia, while IL-4 significantly dampened its expression levels in both IL-4-LV and G93A-IL-4-LV mice (Fig. 7k). *Csf1r* levels were lower in E12.5 and P2 microglia than in adult cells. IL-4 significantly reduced expression levels of this gene in both WT and G93A mice (Fig. 7l). *Selplg (CD162)* is an adhesion molecule involved in early stages of inflammation^70^. It was highly expressed by adult microglia of GFP-LV mice, but significantly down regulated by IL-4 in both IL-4-LV and G93A-IL-4-LV mice (Fig. 7m). *CD14* expression was highly expressed by microglia of G93A-GFP-LV mice. However, *CD14* was significantly reduced by IL-4 in microglia of IL-4-LV mice and above all of G93A-IL-4-LV mice (Fig. 7n). Altogether, these results suggest that IL-4 gene therapy modulates microglia in the early slow-progressing phase of the disease, ameliorating pathological outcomes of G93A mice. However, such modulation does not produce beneficial effects in the fast progressing phase of the disease, as remarkably observed by scoring life survival of G93A mice.

## Discussion

Microglia activation and neuroinflammation are prominent features of ALS^71^ and therapies aimed to modulate microglia activation could be used to revert MNs degeneration. For instance, the replacement of G93A microglia in G93A/PU.1^−/−^ mice with WT microglia induces a less severe disorder^39^. Recently, O’Rourke showed that C9orf72 is relevant for microglia to maintain lysosomal trafficking and cells lacking this gene display altered responses to inflammatory cues^72^. The early G93A microglia display a M2 protective profile, whereas end stage microglia have a M1-like phenotype and contribute to the progressive and neurotoxic phase of the disease^73^. The constitutive ablation of P2X7 in G93A mice worsened the disease and increased MNs loss^74^, while the administration of Brilliant Blue G, a P2X7 inhibitor, before clinical symptoms, reduces “microgliosis”, delays the onset of the disease and improves locomotion in mice^75^. Intriguingly, passive transfer of regulatory T cells in G93A/Rag2^−/−^ mice prolonged life survival of mice and such amelioration seems to depend on IL-4 released by T lymphocytes^76^.

We attempted to skew microglia toward a protective phenotype using IL-4 gene therapy^77,78^. Given the multifunctional action of IL-4, we reasoned that we would have a great advantage of making a deep characterization of IL-4 in WT microglia^79^. By scoring pSTAT6^+^ cells we observed that IL-4 principally acts on microglia population. Because IL-4 and IL-13 can stimulate cells to secrete chemokines (CCL22, CCL17, and CCL23) able to recruit blood cells from the circulation^80–82^, we performed a BMT to demonstrate that blood cells do not infiltrate the CNS of IL-4-LV mice.

Several attempts have been done to find a reliable model for microglia activation, although there is a lack of unambiguous markers to identify microglia exerting either neurotoxic or protective effects in the CNS. Arg1 is classically used to identify M2-polarized microglia^83^, albeit intracellular pathogens induce macrophages to express Arg1 and a classical M1 response^84^. Although we are aware of these limitations, we used Arg1 as well as Fizz1^85^, to identify microglia in which IL-4 pathways were expected to be active. Besides a relative small number of microglia express Arg1, very few Fizz1^+^ cells were observed and the vast majority of them seems to belong to the perivascular macrophages population^50^. Perivascular macrophages, like macrophages of the choroid plexus, subdural meninges and perivascular space, share common markers with microglia, although they may differ from microglia for their capability to respond to IL-4 signaling and possibly for their IL-4Rα levels^83,86^.

Replicating microglia usually generate daughter cells that stay in proximity to each other^87^. IL-4 increases percentages of mitotically active microglia, although cell densities were only slightly modified in IL-4-LV mice. However, microglia incorporating the initial BrdU and the last EdU tracers were usually isolated cells, suggesting that the process of cell division does not terminate. We can prudently read these data considering that IL-4 instructs cells to enter the S-phase but then they are stuck in their ability to proceed along the M-phase. Probably unknown cues avoid an undesirable growth of microglia population, supporting the view that the healthy brain can only accommodate a certain number of microglia^9^.

GSEA revealed a poor concordance between genes differentially regulated by IL-4 in microglia and genes regulated by IL-4 in macrophages^63,82^, further corroborating the idea that microglia activation and macrophages activation are different processes. On the other hand, yolk sac and embryonic microglia^10^ share a large number of genes in common with IL-4 microglia. We could speculate that IL-4 may be instrumental to reestablish in microglia part of a broad genetic program that dominates the developmental microglia.

In G93A mice, IL-4 reduced the levels of pro-inflammatory cytokines including CD14, which interacts with aggregated SOD1 and participates to the noxious activation of microglia in these mice^88^. IL-4 increases locomotion in G93A mice. However, the number of Arg1^+^ microglia declined when the disease accelerates, suggesting that the anti-inflammatory action of IL-4 is not robust enough to contrast ALS neurodegenerative processes. Because, we did not improve MNs cell survival, we could speculate that IL-4 stimulates neuroprotective effects rather than fostering MNs survival. These apparently conflicting results can be explained considering the multifactorial nature of ALS. Since IL-4 induces some pathways that are shared by embryonic microglia, we could speculate that such activation profiles not necessarily imply the capacity of microglia to sustain the entire process of tissue repair.

## Material and methods

### Animals

Mice were maintained under pathogen-free conditions at San Raffaele Hospital mouse facility (Milan, Italy). All efforts were made to minimize animal suffering and to reduce the number of mice used in accordance with the European Communities Council Directive of 24 November 1986 (86/609/EEC). All animal experimental protocols were approved by the Ethics Review Committee for Animal Experimentation of the Italian Ministry of Health. Procedures were performed according to the guidelines of the Institutional Animal Care and Use Committee of the San Raffaele Scientific Institute (protocol number 703/2015PR). Transgenic mutant SOD1 mice carrying the SOD1^G93A^ allele (strain B6SJL-TgN[SOD1-G93A]1GUR) and CAGG-GFP mice^55^ were purchased from Jackson laboratories, while C57BL/6 J used for breeding and experimental purposes were purchased from Charles River (Italy). Mice received multiple injections of the following S-phase tracers: 5-bromo-2-deoxyuridine (BrdU), 5-iodo-2-deoxyuridine (IdU), (Sigma, St. Louis, MO, USA) and 5-ethynyl-2-deoxyuridine (EdU) (Invitrogen, Carlsbad, CA, USA) at the concentration of 100 mg/kg. Starting from day 70, we injected BrdU in mice receiving either GFP or IL-4 lentivirus for consecutive 3 days (three injections per day), while further injections of IdU (three injections per day) were administered starting from 3 days before the sacrifice. At the time of the sacrifice mice received cumulative EdU labeling (100 mg/kg one injection every 3 h) for consecutive 10 h. At the sacrifice, mice were given an overdose of anesthetic drugs and the cerebrospinal fluid (CSF) was collected from the cisterna magna using a glass capillary (Sutter Instruments) pulled with the PC-10 puller (Narishighe). Mice were then transcardially perfused with saline followed by 80/100 ml 4% paraformaldehyde in PBS, pH 7.2 (Sigma). Spinal cords were coronally sliced at ~6-mm thickness and post fixed in 4% paraformaldehyde (Sigma) in PBS, pH 7.2 for 12 h at + 4 °C. Tissues were cryoprotected in PBS/30% Sucrose (Sigma), embedded in OCT inclusion media and stored at −80 °C before processing. Lumbar spinal cords were 12 µM sectioned, labeled and digital images were acquired every 350 µm in a region encompassing 2.5 mm of the spinal cord. Animal cohort numbers were determined by power analysis based on preliminary results or literature precedent, experiments usually required between 3–10 animals per group.

### Motor function

Motor activity of G93A mice receiving GFP or IL-4- LVs and WT littermates was assessed on 80, 90, and 110-days old animals. Mice were habituated for 1 min on a static rotor and 1 min at constant speed (4 rpm) for two times and then tested for motor function over three trials performed over three consecutive days (one per day). Each trial consisted of three test sessions with 15 min interval between sessions. For each session five mice were placed on an accelerating rotor (4–40 rpm) and the latency to fall was recorded, with a maximum limit for individual animal set at 900 s.

### Primary microglia cultures

Primary microglia cultures were obtained from P2 C57Bl/6 mice. Briefly, we removed meninges from each brain in cold KRB medium containing albumin 0.3% (Sigma) and MgCl2 0.04% (Sigma). Cortices were minced and incubated in 10 ml of KRB including Trypsin 0.25 mg/ml (Sigma) for 15 min at 37 °C, then we added 10 ml of complete KRB supplemented with DNase I 0.05 mg/ml (Sigma) and SB-Trypsin inhibitor 0.08 mg/ml (Sigma) to stop the reaction. Cells (3 × 10^6^/T75 flask) were cultured in DMEM (Gibco) supplemented with 10% FBS (Gibco), L-glutamine (1 mM) (Gibco), penicillin (100 U/ml), (Gibco) and streptomycin (100 mg/ml), (Gibco) for 12 days. Microglia were shaken off and 3 × 10^5^ cells plated on glass coverslips. Cells were further washed after 1 h to remove oligodendrocytes progenitors and then treated with recombinant IL-4 (0.08, 1.24, 2.5, 5, 10, 20, and 40 ng/ml), (R&D system) for 12 h. Cultures were paraformaldehyde fixed for 5 min at room temperature and used for immunofluorescence. While, parallel cultures were used to obtain total RNA extracts and used for real time PCR.

### Immunohistochemistry

Sections were washed three times, 5 min each in PBS, and the blockage of nonspecific binding was performed by using the following mix: PBS 1×/ FBS 10%/ BSA 1 mg/ml/ TritonX100 0.1%, for 1 h at room temperature. In the case of BrdU and IdU detections, DNA was depurinated to make epitopes accessible to antibodies using HCl 2 N for 20’. Slides were then rinsed in borate buffer (0.1 M, pH 8.5, Sigma) for 10 min at room temperature. Antibodies were diluted in blocking mix and incubated at +4 °C overnight according manufacturer’s instructions. The following day, sections were rinsed in PBS and fluorescent secondary antibodies—i.e., never deriving from the species from which the primary antibodies are derived—(Alexafluor conjugated) diluted in blocking mix, were applied according to the manufacturer’s instructions. Slides were washed in PBS and incubated in Hoechst 33342 (Sigma) for nuclei counterstaining. When necessary, antigens retrieval was performed by boiling samples in 10 mM sodium citrate (pH 6) for 5 min. The following antibodies and working concentrations were used: rabbit α-Iba1 1:500 (Wako); rabbit α-Arg1 1:100 (Abcam); rabbit α-Fizz1 1:100 (Abcam); rabbit α-ChAt 1:200; rat α-CD206 1:200 (BioLegend); rabbit α-pH3 1:400 (Cell Signaling); goat α-Iba1 1:100 (Abcam); rat α-BrdU 1:500 (Abcam); mouse α-Pan-halogen 1:100 (BD); rat α-F4/80 1:200 (Abcam); rat α-CD31 1:100 (BD); chicken α-GFP 1:500 (Millipore); rabbit α-Cleaved Caspase 3 1:100 (Cell Signaling technology); rabbit α-P-STAT6 1:100 (Cell Signalling); Isolectin B4 (BSI-B4), FITC conjugate 1:100 (Sigma) and click-it EdU alexafluor 546 imaging reagent (Invitrogen). Tyramide Signal Amplification (TSA) from PerkinElmer was used, when appropriate, to improve fluorescent intensity in single and double immunofluorescence. Immunohistochemistry was carried out as previously described^89^. Slices were incubated in Methanol-H_2_O_2_ 3% for 20 min before adding the blocking mix. Antibodies were diluted in blocking mix and incubated at + 4 °C overnight. The following day, sections were washed in PBS three times, and the biotin-conjugated secondary antibody (Vector labs, Milan, Italy) was applied for 2 h. Then sections were washed before adding the avidin-HRP reagent (Vector). Signals were revealed by incubating slices with 3,3′-Diaminobenzidine (DAB, Sigma) solution. Sections from spinal cord injured mice were used to monitor cell death by cleaved caspase 3 and were gently provided by Dr. Cusimano.

### Imaging

Light and fluorescent images were obtained using the Olympus, BX51 with the following objectives: X20 and X40 and equipped with the following cameras: Leica CCD Microscope DFC3000 G and DMC2900. Sixteen-bit images (1296 × 966 pixels) were acquired for each channel and merges were done using Photoshop (Adobe) CS4. Confocal images were obtained using Leica SP8 with X40 objective equipped with super-sensitive HyD detectors. Fluorescence was recorded as square 8-bit images (1024 × 1024 pixels) and stored as separate image stacks for each channel. Alignment of images to obtain largest field of view of spinal cord sections was done applying the automatic stitching of stack images using the Leica dedicated application (Las-X, Leica). Images in figures show the maximal projections of Z-stacks acquired with a 0.7 µm step and pseudo-colored using Las-X software.

### Lentivirus production and injection

Vesicular stomatitis virus-pseudotyped LV generation: the entire coding region of mouse IL-4 was obtained by PCR using published GenBank data NM_021283.2. IL-4 was cloned into the BamHI and SalI site of pCCL.sin.cPPT.PGK.GFP.WPRE construct that was gently provided by L. Naldini (San Raffaele Scientific Institute, Milan, Italy) to create LV-PGK-IL-4. Lentivirus stocks were produced as described^90^. 70 days old wild type C57BL/6 J and G93A mice received either GFP of IL-4 lentivirus through a single injection. In preparation, animals were given an injection of anesthetic, then 10^7^ P.F.U. were diluted in 10 µl and injected within the cisterna magna of mice using a 27-gauge stainless steel needle curved (40°) at 3.5 mm from the tip, so that it was *J*-shaped^78^.

### BMT transplants

6–8 weeks old C57BL/6 males were used as recipients for all bone marrow transplant experiments. Donor mice were transgenic CAGG-GFP and the donors’ age was 4–6 weeks. Recipient mice to be transplanted were exposed to treosulfan according our published methods^56^. 1 month after transplantation, chimerism was evaluated on 100 μl of total blood and mice with a percentage of chimerism > 90% were injected with IL-4 and GFP LVs.

### Magnetic sorting of CD11b^+^ cells & Real time PCR

Cell suspensions were made from IL-4-treated or GFP-treated G93A mice by Neural Tissue Dissociation Kit (P) (Miltenyi Biotec) according to manufacturer’s instructions. Enriched microglia fraction was filtered through a 70 µm strainer and centrifuged over a 37/70% discontinuous Percoll Gradient (GE Healthcare) and mononuclear cells were isolated from the interface^68^. Cells were sorted using CD11b^+^ (Microglia) Microbeads (Miltenyi Biotec). Briefly, microglial cells were magnetically labeled with CD11b^+^ Microbeads and loaded onto a MACS® column (Miltenyi Biotec), which was placed in the magnetic field of a MACS Separator (Miltenyi Biotec). CD11b^+^ and the flow though of the column containing CD11b^−^ cells were collected. Total RNA was extracted from CD11b^+^ and CD11b^−^ cells using the RNeasy Mini Kit (Qiagen) according to the manufacturer’s recommendations including DNase (Promega) digestion. cDNA synthesis was performed by using ThermoScript RT-PCR System (Invitrogen) and Random Hexamer (Invitrogen), according to the manufacturer’s instructions in final volume of 20 µl. The LightCycler 480 System (Roche) and LightCycler 480 SYBR Green I Master Mix (Roche) were used for real-time PCR. Each sample was normalized using the housekeeping gene Histone H3 with the following primers: H3 F: 5′-GGTGAAGAAACCTCATCGTTACAGGCCTGGTAC-3′ H3 R: 5′-CTGCAAAGC ACCAATAGCTGCACTCTGGA AGC-3′. The following specific primers were used for gene expression analysis:

Iba1 F: 5′-GCAGGAAGAGAGGCTGGAGGGGATC-3′;

Iba1 R: 5′-CTCTTCAGCTCTAGGTGGGTCTTCGG-3′;

IL1β F: 5′-CCTGTCCTGTGTAATGAAAGACGG-3′;

IL1β R: 5′-TGTCCTGACCACTGTTGTTTCCC-3′;

Tnfα F: 5′-GCCTCTTCTCATTCCTGCTTGTGGCAG-3′;

Tnfα R: 5′-GACGTGGGCTACAGGCTTGT CACTCG-3′;

Arg1 F: 5′-GCAGCAGCCGCTGGAACCCAG-3′;

Arg1 R: 5′-GTCCCCGTGGTCTCTCACGTC-3′;

Ym1 F: 5′-GATGGCCTCAACCTGGACTGGC-3′;

Ym1 R: 5′-CTGAGACAGTTCAGGGATCTTGTAC-3′;

CD206 F: 5′-CCACTCTATCCACCTTCACTGATG-3′;

CD206 R: 5′-CCTGCTCGTCCACAGTCCACCG-3′;

Fizz1 F: 5′-CTGATGAGACCATAGAGATTATCGTG-3′;

Fizz1 R: 5′-GCACAGGCAGTTGCAAGTATCTCC-3′;

Cdk1F: 5′-GGCTGATTTCGGCCTTGCCAGAG-3′;

Cdk1R: 5′-TGGTGGCCAGTTCTGCAAATATGG-3′;

CxcR2F: 5′-CTGTTCTTTGCCCTGACCTTGCC-3′;

CxcR2R:5′-GCAGGCTAGTAGCAGAACACTGC-3′;

Selplg F: 5′-GGGCCATCCGTGACTCACTTACC-3′;

Selplg R: 5′-AGAAGCCAAGATGAGGATAATCAGC-3′;

P2ry13F: 5′-GACAGGTTCCTCAAGATCATCATGC-3′;

P2ry13R:5′-ACGGATGATGGCGTTGCCTCCTTG-3′;

Csf1R, F:5′-GTTCATTATCCGCAAGGCTAAAGTC-3′

Csf1R, R:5′-CTCCAATTTTATCTGTGGGGGCTC-3′;

CD14: 5′-GGCCCAGTCAGCTAAACTCGCTC-3′;

CD14R: 5′-CTGTTGTAACTGAGATCCAGCACGC-3′;

Data were analyzed with the 2-ΔΔCT method to determine relative changes in gene expression and IL-4 treated mice were compared to controls receiving GFP lentivirus.

### IL-4 determination

Fresh CSF from mice was immediately stored at −80 °C until analyzed using the enzyme-linked immunosorbent assays (ELISA) procedure with OptEIA™ set for mouse IL-4 (BD-Biosciences) according to manufacturer’s instructions. Concentrations of IL-4 were calculated according to a standard curve and expressed as nanograms per milliliter. When concentrations of the cytokine were below the detection threshold, they were assumed to be 0 ng/ml.

### RNA sequencing

Microglia were extracted by flow cytometry, sorting CD45^low^/CD11b^+^ cells from spinal cords of mice. Total RNA was extracted using Micro-RNeasy kit according manufacture’s guidelines. RNA samples were converted into cDNA by using the QuantSeq 3′ mRNA-Seq Reverse (FWD) Library Prep Kit (Lexogen advertise article, Nature Methods 11 2014) according to manufacturer’s instruction to generate compatible libraries for Illumina sequencing. cDNA libraries were assessed using a BioAnalyzer (Agilent Technologies, USA) before 75 bp single end sequencing using an Illumina HiSeq 2500 system based on standard protocols. Raw sequencing reads (FASTQ) were processed individually and mapped to the mouse genome reference version GRCm38 (mm10) from Gencode (M13). The mapping was performed using STAR (v2.5.3a)^91^ using soft clipping and all other parameters set to default values according to recommended data analysis workflow by Lexogen (http://www.lexogen.com/quantseqdataanalysis). Gene abundance was determined using featureCounts^92^ followed by differentially expressed gene analysis using DESeq2^64,93^. The experiment has been submitted to the Gene Expression Omnibus (GSE103607, http://www.ncbi.nlm.nih.gov/geo/query/acc.cgi?acc = GSE103607) at the National Center for Biotechnology Information. Genes were considered differentially expressed when showing a false discovery rate (FDR) of less than 0.05. Gene set enrichment analysis^64^ used GSEA Pre-ranked with default parameters using the log2 Fold Changes calculated by DESeq2 and the sets of published microglia genes^10^. Enrichment plots were built in R. Due to the known fact that GSEA pre-ranked inflates apparent p values due to inter-gene correlations, only *p-*values < 0.001were considered as statistically significant^94^.

### Statistics

Data are expressed as the mean ± standard error of the mean (s.e.m.) or mean ± standard deviation (s.d.) of independent experiments. Normality was assessed in each experiment by applying either Kolmogorov–Smirnov test (with Dallas–Wilkinson–Lille for *p*-value) or D’ Agostino and Pearson omnibus normality test. Comparisons were made using the unpaired *t*-test, one-way or two-way ANOVA tests, followed by Bonferroni’s multiple Comparison test. Non parametric data have been compared using Mann Whitney test. Statistical tests were carried out using PRISM5.01 (GraphPad Software, La Jolla, CA, USA). Value less than 0.05 was considered statistically significant.

## Electronic supplementary material

Supplementary figure 1

Supplementary figure 2

Supplementary figure 3

Supplementary figure 4

Supplementary figure 5

Supplementary figure 6

SupplementaryTable1
