# Supplementary figures and images for: Interleukin 4 modulates microglia homeostasis and attenuates the early slowly progressive phase of amyotrophic lateral sclerosis
Source: Cell Death Dis. 2018 Feb 14;9(2):250. doi: 10.1038/s41419-018-0288-4 (PMC5833860; doi:10.1038/s41419-018-0288-4)

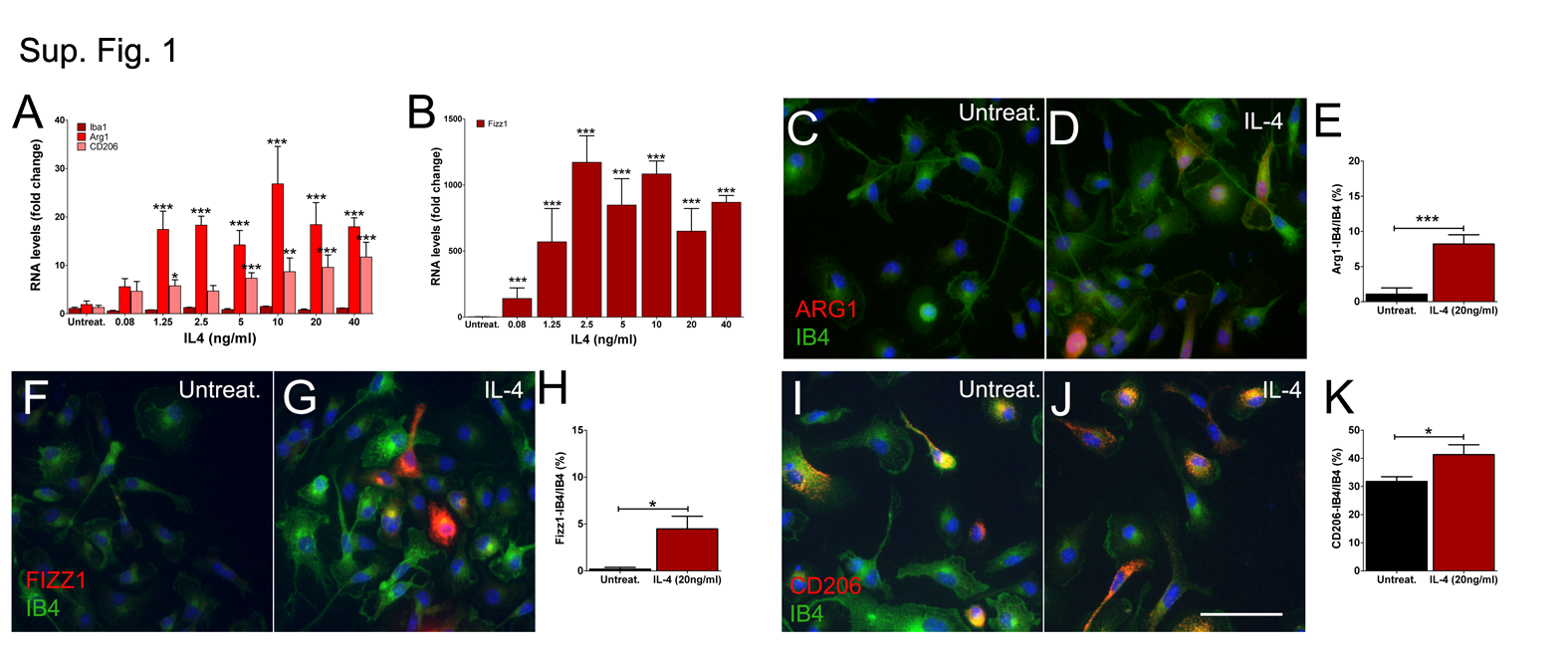

Supplement: Supplementary file 1 — Supplementary figure 1 [file 41419_2018_288_MOESM1_ESM.tif]

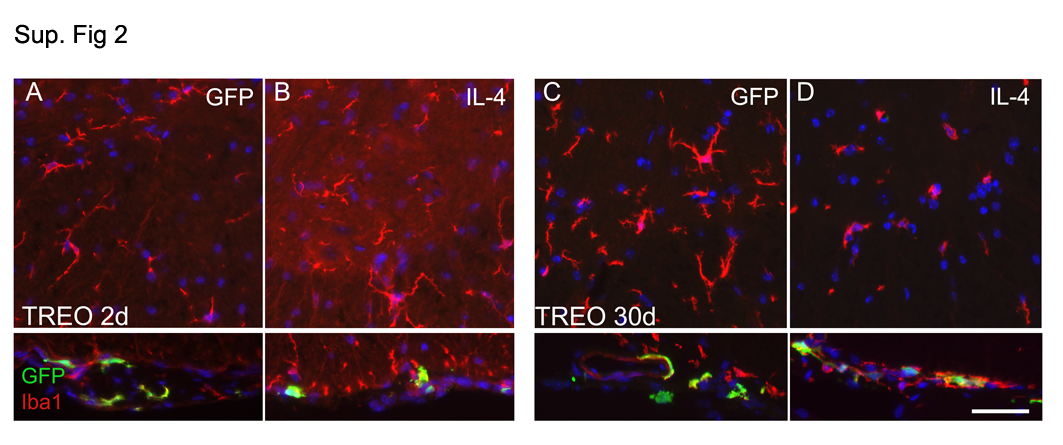

Supplement: Supplementary file 2 — Supplementary figure 2 [file 41419_2018_288_MOESM2_ESM.tif]

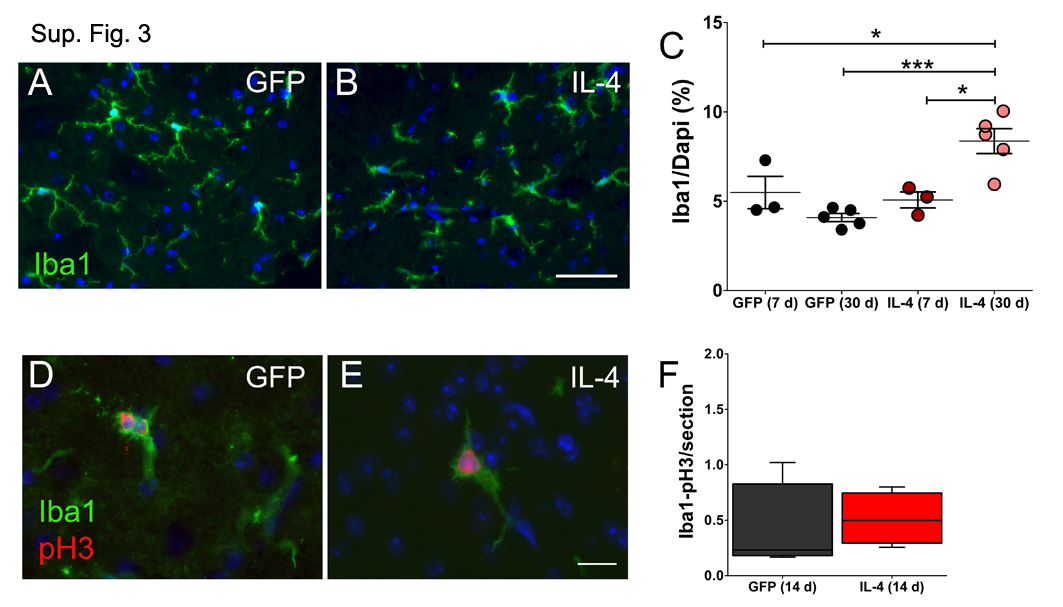

Supplement: Supplementary file 3 — Supplementary figure 3 [file 41419_2018_288_MOESM3_ESM.tif]

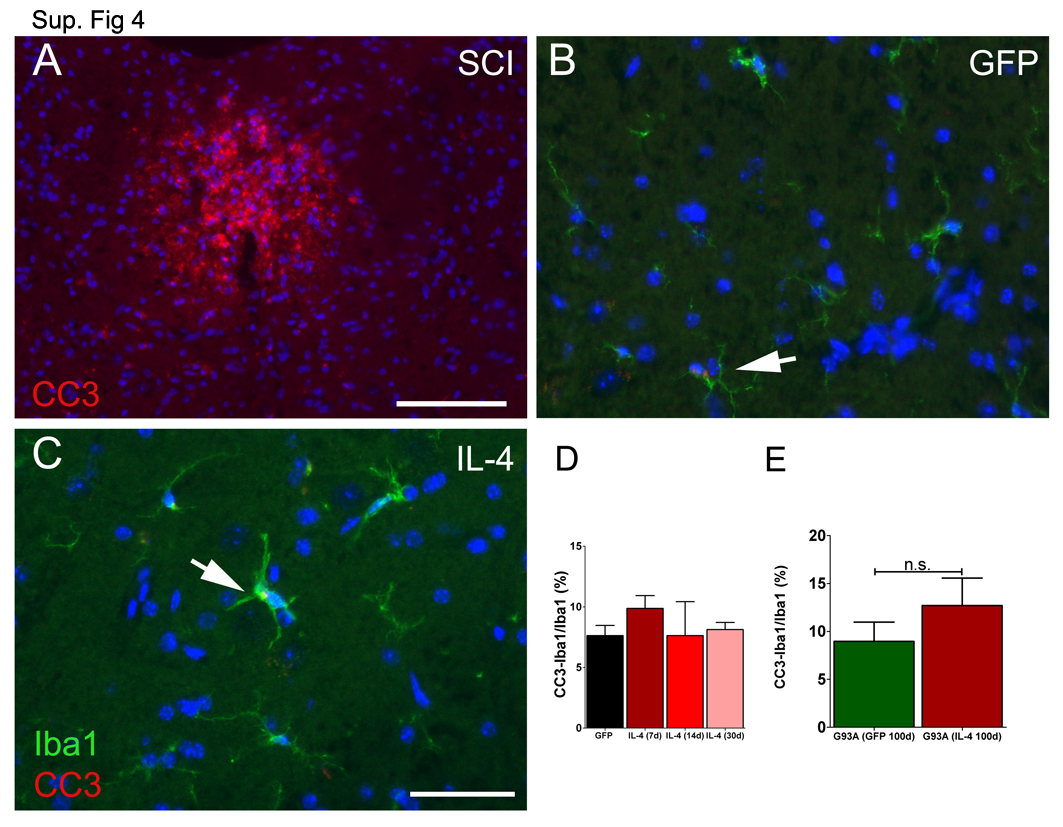

Supplement: Supplementary file 4 — Supplementary figure 4 [file 41419_2018_288_MOESM4_ESM.tif]

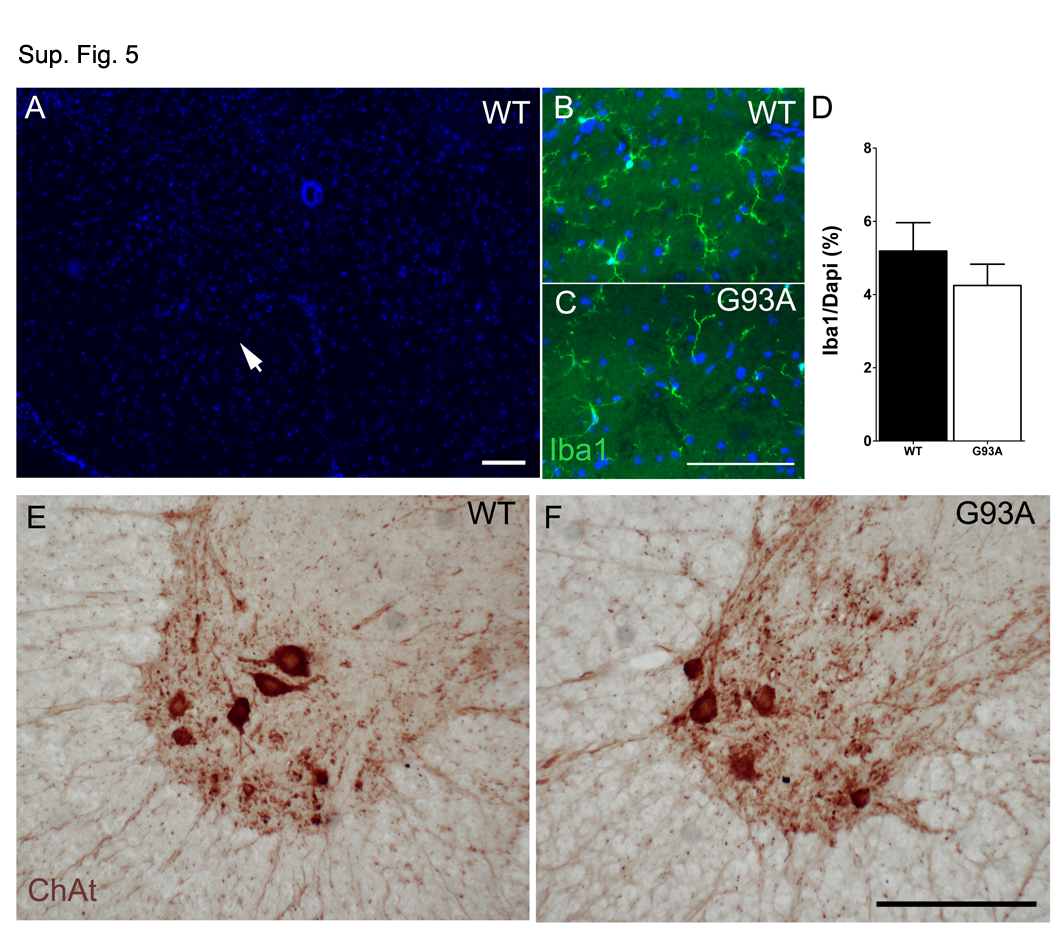

Supplement: Supplementary file 5 — Supplementary figure 5 [file 41419_2018_288_MOESM5_ESM.tif]

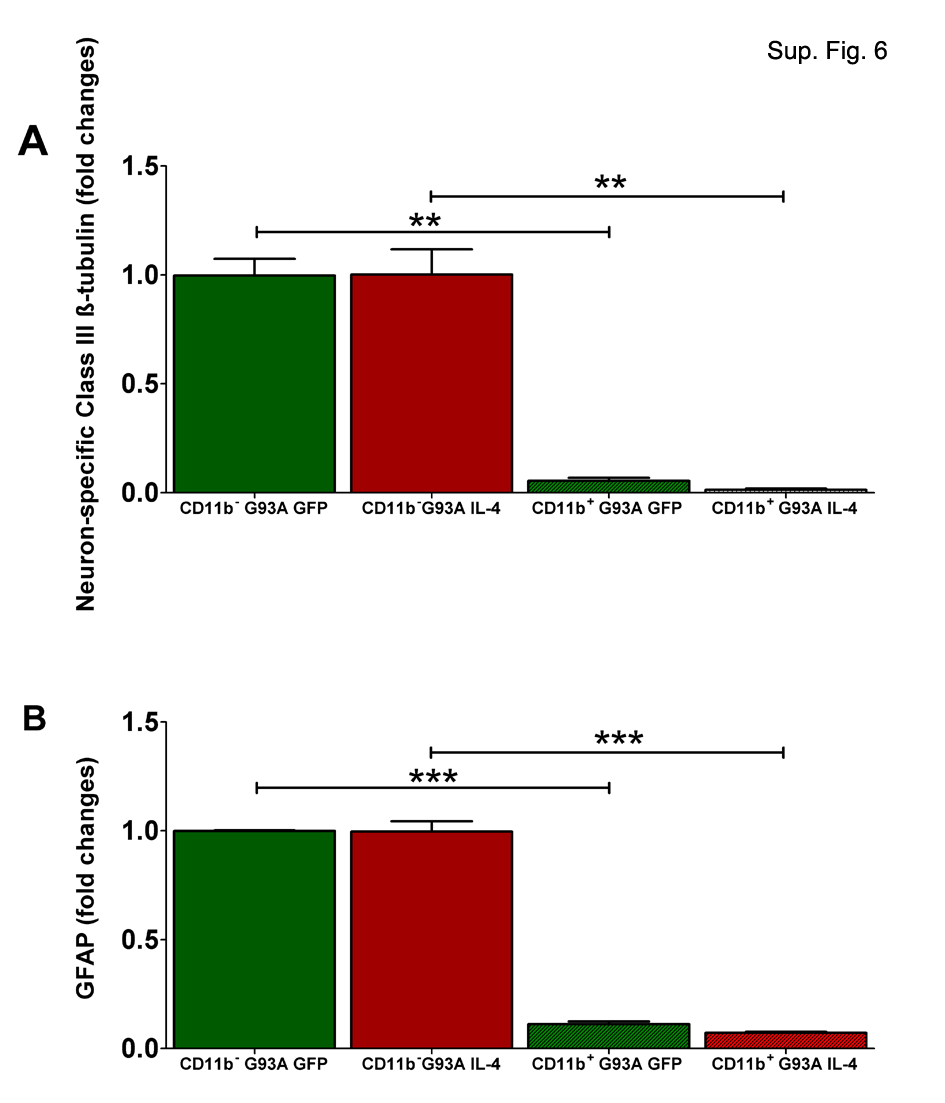

Supplement: Supplementary file 6 — Supplementary figure 6 [file 41419_2018_288_MOESM6_ESM.tif]
