# Supplementary material for: Interleukin 4 modulates microglia homeostasis and attenuates the early slowly progressive phase of amyotrophic lateral sclerosis
Source: Cell Death Dis. 2018 Feb 14;9(2):250. doi: 10.1038/s41419-018-0288-4 (PMC5833860; doi:10.1038/s41419-018-0288-4)
Supplement: Supplementary file 7 — SupplementaryTable1 [file 41419_2018_288_MOESM7_ESM.docx]

| **Table S1. DESeq2 differentially expressed genes in IL4 vs GFP (FDR < 0.05)** | | | | |
| --- | --- | --- | --- | --- |
|  |  |  |  |  |
| **Gene Symbol** | **Mean Expression (Normalized Reads)** | **log2FoldChange(IL4 vs GFP)** | **P.Value** | **FDR** |
| Tmem26 | 175.2449683 | 13.39963388 | 6.91E-07 | 9.58E-05 |
| Ccl24 | 70.81442311 | 12.09089266 | 1.52E-05 | 0.00125656 |
| Rmi2 | 65.11197526 | 11.97165256 | 1.86E-05 | 0.001422652 |
| Lockd | 64.85546317 | 11.96532508 | 1.70E-05 | 0.001358316 |
| Cdkn2a | 61.50721229 | 11.89064151 | 2.03E-05 | 0.001498902 |
| Cdc20 | 52.03381215 | 11.64418873 | 5.59E-05 | 0.003175047 |
| Myh13 | 48.27377215 | 11.54088426 | 4.40E-05 | 0.002748154 |
| Kif4 | 46.69114827 | 11.49314644 | 5.75E-05 | 0.003211581 |
| Aqp3 | 40.9836037 | 11.30307397 | 8.92E-05 | 0.004664524 |
| Cit | 40.95723004 | 11.30077812 | 7.95E-05 | 0.004273207 |
| C330027C09Rik | 40.79495004 | 11.29472951 | 8.28E-05 | 0.004422655 |
| Ccnb1 | 38.26643176 | 11.20366609 | 9.46E-05 | 0.004870961 |
| Eomes | 37.41775296 | 11.17380839 | 0.000376839 | 0.013947227 |
| Ska1 | 36.84695531 | 11.14964594 | 0.000535485 | 0.017837005 |
| Steap3 | 35.81236679 | 11.1095586 | 0.000119084 | 0.005891139 |
| Ncapg | 34.86962493 | 11.06875924 | 0.000136501 | 0.006621634 |
| Fam64a | 32.98536951 | 10.98846473 | 0.000442269 | 0.015345823 |
| Clec4e | 32.5863469 | 10.97551407 | 0.000217486 | 0.009017989 |
| Runx3 | 31.36442218 | 10.91726255 | 0.000189654 | 0.008276044 |
| Pdcd1lg2 | 30.43240713 | 10.87687172 | 0.000229184 | 0.009424829 |
| E2f7 | 28.13789919 | 10.76073076 | 0.000252572 | 0.010136343 |
| Cep55 | 26.99627066 | 10.70100798 | 0.000297559 | 0.011525214 |
| Acp5 | 27.01908368 | 10.70047701 | 0.000737205 | 0.021912203 |
| Zfp641 | 26.34438578 | 10.66815231 | 0.000979012 | 0.026369985 |
| Prr11 | 26.12482957 | 10.65502015 | 0.000319438 | 0.012183743 |
| Ccna2 | 24.12630559 | 10.53724105 | 0.000424359 | 0.01503765 |
| Cdca5 | 23.88897419 | 10.52702587 | 0.000427442 | 0.015044829 |
| Ect2 | 23.18281175 | 10.48018619 | 0.000468136 | 0.015966158 |
| F10 | 22.47685043 | 10.43695154 | 0.000499875 | 0.01681902 |
| Pqlc3 | 21.51000785 | 10.37764435 | 0.000786379 | 0.022388265 |
| Nynrin | 20.7524447 | 10.32246784 | 0.000661656 | 0.020219953 |
| Cdc6 | 20.45497772 | 10.30170749 | 0.000670965 | 0.020379787 |
| Ccdc18 | 20.33475963 | 10.29182402 | 0.001506128 | 0.035884547 |
| Gm37006 | 20.20997123 | 10.28055806 | 0.000773173 | 0.022354028 |
| Gm6169 | 19.58281143 | 10.23947747 | 0.00077593 | 0.022354028 |
| Unc5cl | 17.99343296 | 10.11455845 | 0.001639792 | 0.037669971 |
| Nek2 | 17.36570186 | 10.06674433 | 0.00149048 | 0.03566699 |
| Cdkn3 | 17.14104545 | 10.04641841 | 0.00115875 | 0.029690734 |
| Dtl | 16.76870777 | 10.01457736 | 0.001771216 | 0.039332801 |
| Arg1 | 4906.475622 | 10.00771767 | 1.20E-126 | 1.20E-122 |
| Stil | 16.60916586 | 9.997090327 | 0.001465574 | 0.035290316 |
| Rbks | 16.49448869 | 9.994163901 | 0.00153956 | 0.036153694 |
| Mtfr2 | 16.20169868 | 9.962761215 | 0.001714059 | 0.038491214 |
| Adgrg3 | 15.89882955 | 9.939914225 | 0.001693783 | 0.038352834 |
| Gm23296 | 15.4304748 | 9.898589803 | 0.001948174 | 0.042138751 |
| Acy1 | 15.2092461 | 9.873993909 | 0.001722865 | 0.038602215 |
| Slc7a2 | 275.0021947 | 9.862324487 | 1.85E-16 | 1.54E-13 |
| Gm8113 | 131.988947 | 9.847210179 | 8.92E-10 | 3.43E-07 |
| Rps11-ps3 | 14.72519144 | 9.828137285 | 0.002121285 | 0.043714411 |
| Ear2 | 14.71764927 | 9.827908824 | 0.002277021 | 0.045417698 |
| Rnase2a | 1581.932727 | 9.827189869 | 1.10E-76 | 5.48E-73 |
| Pold1 | 14.67439466 | 9.818545019 | 0.002128366 | 0.043714411 |
| Neil3 | 14.3756726 | 9.789937309 | 0.002044463 | 0.042920833 |
| Gm43863 | 14.32789491 | 9.787281419 | 0.002005722 | 0.042645062 |
| Cfp | 14.30286203 | 9.784948784 | 0.002156478 | 0.044159184 |
| Bst1 | 14.15924797 | 9.766420635 | 0.002344231 | 0.046479959 |
| Cdca8 | 121.8599065 | 9.711318555 | 3.17E-09 | 1.13E-06 |
| Dmrtb1 | 186.5336503 | 8.742831463 | 8.24E-18 | 8.23E-15 |
| Retnla | 660.4560514 | 8.657072764 | 2.06E-47 | 6.85E-44 |
| Prkaa2 | 58.49452162 | 8.655873698 | 6.78E-07 | 9.58E-05 |
| Serpinb6b | 56.82841925 | 8.615669508 | 2.30E-06 | 0.000270865 |
| Cenpf | 157.0973797 | 8.557666008 | 2.55E-12 | 1.41E-09 |
| Knstrn | 52.02850801 | 8.485146994 | 1.19E-06 | 0.000154266 |
| Hmgb3 | 47.63553058 | 8.36246056 | 1.76E-05 | 0.001362745 |
| Snord22 | 40.61824876 | 8.182219219 | 3.13E-05 | 0.002033111 |
| AI661453 | 40.04457077 | 8.152838089 | 1.34E-05 | 0.001146675 |
| Gm42835 | 41.34172952 | 8.074147505 | 7.70E-06 | 0.000718832 |
| C730034F03Rik | 37.01822845 | 7.992440881 | 1.37E-05 | 0.001154452 |
| Esco2 | 36.2414485 | 7.967195397 | 4.54E-05 | 0.002789675 |
| Msx3 | 99.17056253 | 7.852057228 | 1.47E-11 | 7.73E-09 |
| Melk | 32.65349299 | 7.808957573 | 5.75E-05 | 0.003211581 |
| Eri2 | 28.11616715 | 7.670085993 | 0.000705506 | 0.021171533 |
| Hist1h1b | 31.50127206 | 7.661992452 | 4.93E-05 | 0.002947418 |
| Mki67 | 397.3700352 | 7.330340085 | 2.29E-23 | 3.81E-20 |
| Prdm16 | 20.93501898 | 7.230238277 | 0.000641334 | 0.019780415 |
| Emilin2 | 66.88414452 | 7.216580115 | 1.20E-08 | 3.53E-06 |
| Gm45716 | 23.21489456 | 7.200918946 | 0.000411683 | 0.01479836 |
| Hacd1 | 19.9824322 | 7.10718604 | 0.001173044 | 0.029903641 |
| Sgol1 | 39.17739906 | 7.080636401 | 2.04E-05 | 0.001498902 |
| Glipr2 | 19.56924924 | 7.078901408 | 0.001657454 | 0.037814921 |
| Foxm1 | 69.66767473 | 6.989821065 | 1.27E-08 | 3.53E-06 |
| Prc1 | 267.0106085 | 6.932987799 | 6.23E-19 | 6.91E-16 |
| Asf1b | 32.913548 | 6.899750132 | 2.72E-05 | 0.001825042 |
| Shq1 | 17.3800173 | 6.899067322 | 0.001568615 | 0.036577478 |
| Bmp2 | 16.4360287 | 6.891927776 | 0.001935642 | 0.042049711 |
| Spock1 | 285.3353891 | 6.804641805 | 7.20E-27 | 1.80E-23 |
| Ccne2 | 30.90783786 | 6.783638409 | 0.000814912 | 0.02306917 |
| Kynu | 16.94523066 | 6.720252563 | 0.002305862 | 0.045901359 |
| Gm24357 | 16.86612718 | 6.716481512 | 0.001964066 | 0.042208406 |
| 1700037H04Rik | 27.61877194 | 6.654161767 | 0.00016026 | 0.007346218 |
| Nuf2 | 29.1600273 | 6.650776782 | 0.00040761 | 0.014704853 |
| Tmem51 | 175.8829065 | 6.592119317 | 5.88E-19 | 6.91E-16 |
| Gm11686 | 26.83744893 | 6.563898009 | 5.02E-05 | 0.002966551 |
| Trabd2b | 115.9330174 | 6.446209006 | 6.85E-09 | 2.28E-06 |
| Cish | 106.6363123 | 6.309150922 | 4.23E-09 | 1.46E-06 |
| Rhof | 20.9317366 | 6.258609977 | 0.000785697 | 0.022388265 |
| Ass1 | 21.63788054 | 6.159698995 | 0.001326968 | 0.032986036 |
| Sestd1 | 46.49331457 | 6.076272577 | 1.22E-06 | 0.00015506 |
| Adgre5 | 426.0048313 | 6.062287817 | 2.81E-14 | 2.01E-11 |
| Mir5136 | 25.91101068 | 5.99537908 | 0.000433847 | 0.015158849 |
| Gm24305 | 39.31765941 | 5.941575871 | 3.87E-05 | 0.002455237 |
| Zfp938 | 16.65854281 | 5.883413268 | 0.001767285 | 0.039332801 |
| Cp | 296.5499721 | 5.881042631 | 4.00E-15 | 3.07E-12 |
| Aldh1l2 | 22.44180818 | 5.740536541 | 0.000369607 | 0.013781666 |
| Rpl36a-ps1 | 16.1745963 | 5.739945411 | 0.002450723 | 0.047738928 |
| Kbtbd11 | 38.01870563 | 5.726617754 | 5.41E-05 | 0.003126667 |
| Postn | 264.726631 | 5.719170319 | 0.001094529 | 0.028707698 |
| Prrg1 | 21.44885877 | 5.643439495 | 0.00075884 | 0.02218136 |
| Flt1 | 2373.666909 | 5.527991568 | 4.27E-17 | 3.88E-14 |
| Itgal | 77.34700375 | 5.472775181 | 5.34E-05 | 0.003105008 |
| Poc1a | 20.1755854 | 5.442811633 | 0.001711276 | 0.038491214 |
| Kyat1 | 22.64096145 | 5.350722257 | 0.000605902 | 0.019152336 |
| Igf1 | 1263.802567 | 5.29098293 | 5.83E-05 | 0.003236332 |
| Cenpe | 193.937769 | 5.278223098 | 3.35E-07 | 5.31E-05 |
| Nusap1 | 217.0860768 | 5.238008692 | 9.51E-08 | 1.79E-05 |
| Ldlrad3 | 33.20495193 | 5.197741203 | 0.001781894 | 0.039482197 |
| AF529169 | 451.5690516 | 5.13071814 | 1.01E-10 | 4.81E-08 |
| Fgf11 | 34.68986787 | 5.080533821 | 0.001428253 | 0.034811057 |
| Tmem156 | 53.92265943 | 5.078478162 | 7.50E-05 | 0.004072206 |
| Gmnn | 79.33386294 | 5.063968763 | 0.00014558 | 0.006862159 |
| Ung | 39.70788928 | 5.049775336 | 0.000741151 | 0.021912203 |
| Ptpn7 | 33.74023657 | 4.863231269 | 0.000611132 | 0.019204522 |
| Nmb | 21.39177765 | 4.831025064 | 0.00111979 | 0.029140784 |
| Mfge8 | 332.1085109 | 4.772710746 | 1.03E-08 | 3.20E-06 |
| Raph1 | 25.542716 | 4.692933062 | 0.001235717 | 0.031104597 |
| Timeless | 35.09627163 | 4.555321086 | 0.001754379 | 0.039140335 |
| Birc5 | 243.8166513 | 4.527081619 | 2.09E-06 | 0.000252162 |
| Kif22 | 55.21530117 | 4.526034148 | 0.002066509 | 0.042949843 |
| Cracr2a | 49.82616809 | 4.461527027 | 2.78E-05 | 0.001852178 |
| I830127L07Rik | 36.70905111 | 4.456387254 | 0.001196215 | 0.030262721 |
| Gins2 | 95.42937386 | 4.427654055 | 1.96E-05 | 0.0014862 |
| Cdk1 | 196.4420426 | 4.379481319 | 2.94E-06 | 0.000333623 |
| Gm37298 | 27.78426396 | 4.328855233 | 0.002438083 | 0.047660703 |
| Gm28438 | 35.35620308 | 4.324619803 | 0.002589823 | 0.049961582 |
| Olfm1 | 38.7400048 | 4.239311881 | 0.001440883 | 0.034952443 |
| Xdh | 760.1726394 | 4.085853032 | 1.16E-07 | 2.12E-05 |
| Psma5 | 69.38757293 | 4.071072664 | 0.000804616 | 0.022842406 |
| C3 | 1647.307688 | 4.048304564 | 1.05E-12 | 6.54E-10 |
| Ska2 | 34.6993632 | 4.017220406 | 0.001975677 | 0.042366814 |
| Ero1l | 72.61505523 | 4.012338369 | 0.000890286 | 0.024441279 |
| Inf2 | 81.82791237 | 3.956548564 | 6.82E-05 | 0.003745053 |
| Cd300lb | 73.86802097 | 3.854452798 | 0.0014113 | 0.034566464 |
| Cox6a2 | 497.7035214 | 3.723931435 | 2.98E-09 | 1.10E-06 |
| St8sia1 | 226.2328181 | 3.703658998 | 0.000142273 | 0.00680255 |
| Top2a | 919.0611477 | 3.638803311 | 4.35E-08 | 9.88E-06 |
| Cd36 | 175.9281563 | 3.503221215 | 0.000818757 | 0.02311255 |
| Cd40 | 101.6243724 | 3.479909446 | 0.001080874 | 0.028574536 |
| Cks1b | 192.5509059 | 3.477616877 | 0.000141717 | 0.00680255 |
| Anxa2 | 587.7315253 | 3.470478539 | 1.76E-08 | 4.40E-06 |
| Gm26205 | 85.27242383 | 3.465093164 | 0.002197145 | 0.044445478 |
| Ndufa1 | 853.8308125 | 3.398006548 | 1.62E-05 | 0.001313085 |
| Iqgap3 | 45.38459567 | 3.322593538 | 0.000991193 | 0.026626334 |
| Mmd | 140.8478645 | 3.253633019 | 0.001682096 | 0.038202685 |
| Anxa4 | 208.4064806 | 3.219154326 | 4.26E-06 | 0.000468149 |
| Iqgap1 | 304.7998019 | 3.188630636 | 0.000414444 | 0.014839369 |
| Tpx2 | 107.3824287 | 3.153619559 | 0.000766453 | 0.022329926 |
| Rnaseh2c | 77.98868371 | 3.112005947 | 0.002060276 | 0.042949843 |
| Gatm | 661.6922316 | 3.086982595 | 1.81E-13 | 1.20E-10 |
| Vegfa | 109.7070507 | 3.05728565 | 0.001578081 | 0.036620529 |
| Stmn1 | 410.9089583 | 3.028495934 | 4.91E-08 | 1.07E-05 |
| Rpl21-ps8 | 132.0890866 | 2.972104922 | 0.001491925 | 0.03566699 |
| Nfil3 | 405.6025915 | 2.959825668 | 1.42E-07 | 2.50E-05 |
| Cyp4f18 | 107.9228689 | 2.951017976 | 0.000523555 | 0.017497933 |
| Uqcrq | 393.8976384 | 2.935772708 | 0.000708098 | 0.021185712 |
| Smc2 | 451.4648456 | 2.910819053 | 0.000191443 | 0.008317802 |
| Cd72 | 387.2114039 | 2.747517278 | 0.000427572 | 0.015044829 |
| Wfdc21 | 327.7927347 | 2.741684733 | 0.002031316 | 0.042920833 |
| Rpl39 | 596.7556774 | 2.735193299 | 9.84E-05 | 0.004989134 |
| Glrx | 106.5091535 | 2.688387974 | 0.000216848 | 0.009017989 |
| Rpl35 | 1678.796998 | 2.683289243 | 1.97E-07 | 3.33E-05 |
| Atp5j | 248.120995 | 2.678830666 | 6.00E-06 | 0.000611393 |
| Clec4a3 | 425.6255167 | 2.661257824 | 0.002494156 | 0.048396304 |
| Prps1 | 757.3555829 | 2.641652748 | 2.32E-10 | 9.66E-08 |
| Sdc4 | 1321.115996 | 2.624976707 | 5.34E-08 | 1.13E-05 |
| Ndufa13 | 465.8249296 | 2.601868535 | 3.23E-05 | 0.002080365 |
| Atf5 | 961.2639337 | 2.585090126 | 1.11E-10 | 5.05E-08 |
| Col6a3 | 1146.290748 | 2.564363642 | 0.000239966 | 0.009787662 |
| Cd74 | 795.8199019 | 2.513071218 | 5.31E-05 | 0.003105008 |
| Ak2 | 926.7007791 | 2.492034265 | 2.49E-08 | 5.91E-06 |
| Gpr65 | 683.5392162 | 2.479990061 | 5.56E-10 | 2.22E-07 |
| Txn1 | 344.0000697 | 2.425808314 | 0.000844909 | 0.02367092 |
| Rpl30 | 1365.466147 | 2.422439417 | 5.61E-06 | 0.000577616 |
| Eml2 | 295.3998511 | 2.418993898 | 0.000313444 | 0.012005567 |
| Rpl28 | 1034.22973 | 2.413873819 | 4.65E-07 | 7.26E-05 |
| Rps16 | 2297.196769 | 2.406818712 | 9.85E-07 | 0.00012955 |
| Rpl38 | 2189.325533 | 2.395462385 | 1.14E-05 | 0.001019265 |
| Tbc1d4 | 75.02266509 | 2.391511408 | 0.001100314 | 0.028783862 |
| Zfp609 | 306.8897855 | 2.385907262 | 0.001309643 | 0.03263656 |
| Gsn | 81.19612913 | 2.365364128 | 0.002262316 | 0.045331619 |
| S100a6 | 544.6187584 | 2.355947231 | 0.001105833 | 0.028852713 |
| Rpl41 | 1566.891708 | 2.342882236 | 2.26E-07 | 3.70E-05 |
| Rps27a | 1544.412089 | 2.338519334 | 6.35E-06 | 0.000634671 |
| Rpl10a | 251.1136423 | 2.337442067 | 0.0022409 | 0.045185351 |
| Gm14303 | 116.8574497 | 2.317410597 | 0.000554737 | 0.018122446 |
| Atp5o | 321.7160095 | 2.242996576 | 1.88E-06 | 0.000228838 |
| Hes1 | 835.3454926 | 2.170235634 | 0.000195289 | 0.00841175 |
| Fcgrt | 337.5784221 | 2.169064627 | 1.16E-07 | 2.12E-05 |
| Rpl35a | 685.2065122 | 2.145589604 | 0.000126453 | 0.006194355 |
| Sh3bgrl3 | 515.791166 | 2.132680819 | 1.80E-05 | 0.001385226 |
| Eif2b2 | 209.1404074 | 2.125454349 | 0.001149869 | 0.029631271 |
| Bola2 | 210.3197672 | 2.123699941 | 0.001377859 | 0.033913654 |
| Rpl37a | 5327.868914 | 2.120123126 | 9.85E-07 | 0.00012955 |
| Cst7 | 639.2431852 | 2.104509558 | 6.80E-05 | 0.003745053 |
| Cox8a | 1405.622536 | 2.084337625 | 2.21E-06 | 0.000262828 |
| Neurl1b | 276.7420496 | 2.07357622 | 0.000259977 | 0.010389221 |
| Ezr | 323.6709922 | 2.073171268 | 0.000174414 | 0.007851 |
| Hltf | 891.8416065 | 2.062785173 | 0.000429568 | 0.015062008 |
| Clybl | 269.2929479 | 2.050887952 | 0.000555946 | 0.018122446 |
| Rps8 | 4799.15383 | 2.039835312 | 5.10E-06 | 0.000542703 |
| Tmem258 | 791.5217345 | 2.017467132 | 6.86E-07 | 9.58E-05 |
| Rpf2 | 133.0940128 | 1.995907503 | 0.001308956 | 0.03263656 |
| Rps24 | 1941.620536 | 1.975456288 | 2.67E-07 | 4.31E-05 |
| Rbms1 | 485.4258785 | 1.96655658 | 4.34E-05 | 0.002728667 |
| Abcd2 | 917.5398104 | 1.956571948 | 2.52E-05 | 0.001740265 |
| Gm1966 | 626.6744081 | 1.940938009 | 4.73E-07 | 7.26E-05 |
| Pcyox1l | 1238.414533 | 1.940278735 | 2.85E-05 | 0.001888793 |
| Cd300lf | 720.2150116 | 1.930034625 | 8.80E-05 | 0.004627154 |
| Minos1 | 191.8791606 | 1.921056238 | 0.000777735 | 0.022354028 |
| Cox6c | 1332.388915 | 1.907838631 | 1.63E-11 | 8.14E-09 |
| Rpl32 | 1230.519023 | 1.899812261 | 1.19E-05 | 0.00105358 |
| Rpl36al | 242.1043663 | 1.897234434 | 0.00113396 | 0.029356632 |
| Igbp1 | 573.6322238 | 1.894764655 | 4.98E-05 | 0.002963986 |
| Anapc13 | 560.433496 | 1.892735924 | 8.48E-05 | 0.004482016 |
| Rpl5 | 805.6320556 | 1.876364415 | 9.28E-05 | 0.004807504 |
| Add3 | 364.0618658 | 1.868765707 | 0.000313565 | 0.012005567 |
| Dpm3 | 227.4578321 | 1.842733454 | 0.000863181 | 0.023960455 |
| mt-Nd6 | 446.2454317 | 1.842425808 | 0.002263632 | 0.045331619 |
| Rps11 | 1198.900752 | 1.835123238 | 2.11E-05 | 0.001528348 |
| Cops9 | 970.3503203 | 1.810125886 | 2.25E-05 | 0.001584092 |
| Baiap2 | 201.140533 | 1.798938029 | 0.001654729 | 0.037814921 |
| Vrk1 | 510.7738717 | 1.794656455 | 0.002116316 | 0.043714411 |
| Fau | 2474.011296 | 1.794235481 | 2.69E-06 | 0.000313019 |
| Psap | 15217.90058 | 1.78696489 | 5.69E-07 | 8.48E-05 |
| Lyz2 | 6917.954692 | 1.77930588 | 1.86E-08 | 4.53E-06 |
| Rps2 | 421.3746575 | 1.774249463 | 0.000565714 | 0.018295091 |
| Ndufa5 | 365.892285 | 1.767010943 | 0.000872053 | 0.02405763 |
| Rps15 | 2277.885827 | 1.760859395 | 1.47E-08 | 3.88E-06 |
| Tomm7 | 1040.849238 | 1.759793049 | 0.001984306 | 0.04246074 |
| Rps5 | 3028.685301 | 1.735979087 | 1.04E-05 | 0.000931893 |
| Rps21 | 15197.14515 | 1.725030266 | 2.14E-05 | 0.001528348 |
| Oaz2 | 329.5614633 | 1.717994034 | 0.000559776 | 0.018161833 |
| Rps27 | 2294.224159 | 1.714309043 | 0.001696383 | 0.038352834 |
| Lig1 | 411.8187848 | 1.68821238 | 4.85E-05 | 0.002921904 |
| Ifi30 | 774.4196897 | 1.687514634 | 0.00025082 | 0.010106618 |
| Rpl36a | 7610.73555 | 1.676039591 | 1.93E-10 | 8.38E-08 |
| Rpl23 | 9029.919584 | 1.673220538 | 2.62E-08 | 6.09E-06 |
| Rps28 | 3363.239188 | 1.672547987 | 5.46E-06 | 0.000568745 |
| Sys1 | 281.6329141 | 1.648693637 | 0.00046556 | 0.015966158 |
| Ppp2r3a | 292.8553039 | 1.640403845 | 0.001457939 | 0.035191274 |
| Rps7 | 1865.969891 | 1.619547629 | 9.27E-06 | 0.000842229 |
| Pde4dip | 459.8554117 | 1.615253104 | 0.000553419 | 0.018122446 |
| Diaph1 | 377.6828832 | 1.606642729 | 0.000374044 | 0.013895245 |
| Rpl27 | 4188.695004 | 1.603670456 | 0.000180892 | 0.008069886 |
| Uqcrh | 1708.280272 | 1.586659682 | 2.75E-06 | 0.0003157 |
| Ndufb8 | 537.5913157 | 1.586470138 | 2.25E-05 | 0.001584092 |
| Rpl14 | 1392.256645 | 1.581644509 | 2.06E-05 | 0.001505303 |
| Rps14 | 5174.38917 | 1.57067167 | 1.51E-05 | 0.00125656 |
| Rpl37 | 8022.432219 | 1.563992868 | 1.88E-07 | 3.24E-05 |
| Sorl1 | 449.7634031 | 1.562577294 | 0.000484216 | 0.01640261 |
| Prdx5 | 599.2586955 | 1.562233717 | 0.000109187 | 0.005455504 |
| Rpl34 | 2299.822381 | 1.554540316 | 0.000175777 | 0.007876861 |
| Rnase4 | 2443.076917 | 1.546939134 | 4.65E-05 | 0.002835165 |
| Slc25a4 | 1209.938416 | 1.512413096 | 0.000521121 | 0.01747505 |
| Atp5e | 574.5749242 | 1.508024868 | 0.000199581 | 0.008532021 |
| Clec7a | 2164.237776 | 1.507125748 | 6.68E-06 | 0.000654367 |
| mt-Nd5 | 3102.471197 | 1.500998013 | 1.36E-05 | 0.001154382 |
| Bcl2a1b | 1171.3478 | 1.493245622 | 0.000288405 | 0.011298248 |
| 2010107E04Rik | 563.5619694 | 1.481895241 | 0.000153578 | 0.007170609 |
| Eid1 | 377.7953831 | 1.479945292 | 0.000143468 | 0.006804485 |
| Cd52 | 1593.609809 | 1.469223082 | 3.13E-05 | 0.002033111 |
| Rps20 | 5637.626795 | 1.468137543 | 1.62E-06 | 0.00019983 |
| Atp5g3 | 2511.900135 | 1.465529699 | 9.83E-05 | 0.004989134 |
| Rps19 | 4800.075927 | 1.458175194 | 7.53E-07 | 0.000103085 |
| Usp1 | 350.920948 | 1.446253069 | 0.000607554 | 0.019152336 |
| Ppib | 428.9088388 | 1.445361528 | 0.000394049 | 0.014267132 |
| Rpl17 | 842.6085058 | 1.43652353 | 0.001180053 | 0.029929618 |
| Rpl31 | 2788.748458 | 1.43438515 | 4.50E-05 | 0.002789675 |
| Myo5a | 817.4928474 | 1.424444617 | 0.000684906 | 0.020740193 |
| Rplp2 | 13637.7653 | 1.412346677 | 1.50E-08 | 3.88E-06 |
| Csf2ra | 666.1067479 | 1.410686654 | 0.000588028 | 0.01877368 |
| Trpm2 | 892.6816445 | 1.403905684 | 1.34E-05 | 0.001146675 |
| Rplp1 | 15260.0891 | 1.394918927 | 1.52E-08 | 3.88E-06 |
| Naca | 766.2573849 | 1.387982783 | 4.70E-05 | 0.002844838 |
| Rps3a1 | 563.6681583 | 1.386662465 | 0.001639205 | 0.037669971 |
| Rps12 | 12168.0669 | 1.380441118 | 2.11E-07 | 3.52E-05 |
| Rplp0 | 3816.851887 | 1.376575625 | 0.00021234 | 0.008943203 |
| Dhrs7 | 508.3549147 | 1.353993421 | 0.002174741 | 0.044175425 |
| Rpsa | 1034.224103 | 1.345746654 | 2.29E-05 | 0.001598916 |
| Rpl7 | 1415.421799 | 1.335822315 | 7.54E-06 | 0.000711176 |
| Serpine1 | 1787.610263 | 1.329900704 | 2.02E-05 | 0.001498902 |
| Rack1 | 3630.558467 | 1.32680563 | 0.000368888 | 0.013781666 |
| Il1rl2 | 371.91224 | 1.324200078 | 0.002380016 | 0.046817906 |
| Rpl6 | 1784.853693 | 1.322144131 | 0.000102303 | 0.005137266 |
| Rpl26 | 3760.263642 | 1.32108712 | 7.39E-06 | 0.000703379 |
| Ndufb9 | 553.2753083 | 1.315557378 | 0.001058157 | 0.02819778 |
| Tgm2 | 1414.558064 | 1.309323991 | 0.00039233 | 0.014256545 |
| Rps17 | 1605.473595 | 1.308412343 | 0.001655116 | 0.037814921 |
| Eef1b2 | 4055.017692 | 1.306947812 | 7.87E-08 | 1.57E-05 |
| Rpl21 | 2679.387655 | 1.300202902 | 0.000848013 | 0.02367092 |
| Gas5 | 4901.753955 | 1.296759228 | 0.000171624 | 0.007760349 |
| Ndufa6 | 621.3886519 | 1.291779254 | 0.000556749 | 0.018122446 |
| mt-Cytb | 6826.585823 | 1.281492513 | 0.000212997 | 0.008943203 |
| Rpl13 | 2432.184871 | 1.260561865 | 5.50E-05 | 0.003140966 |
| Rpl9 | 1752.033471 | 1.259006927 | 0.00049395 | 0.016675807 |
| Rps3 | 4021.695674 | 1.250214388 | 1.75E-05 | 0.001362745 |
| Lmo4 | 792.2479231 | 1.220895371 | 0.002490132 | 0.048396304 |
| Anxa5 | 353.1014638 | 1.213145376 | 0.002423847 | 0.047493137 |
| Rpl3 | 1655.577641 | 1.210845549 | 0.00122399 | 0.030887207 |
| Rps29 | 8928.940442 | 1.207825798 | 8.57E-06 | 0.000785489 |
| Rps13 | 3123.492022 | 1.179745255 | 0.000234774 | 0.009615141 |
| Sars | 907.1638019 | 1.173068508 | 0.00235784 | 0.046565008 |
| Rassf3 | 440.1948734 | 1.158163995 | 0.001994523 | 0.042588176 |
| Rpl22 | 3767.005416 | 1.134448002 | 0.000304557 | 0.011750721 |
| Chic2 | 802.8156125 | 1.127337558 | 0.000466808 | 0.015966158 |
| Rpl27a | 1896.080083 | 1.119450724 | 0.000279763 | 0.011050075 |
| AU020206 | 2634.675758 | 1.107268584 | 6.99E-06 | 0.000672216 |
| Atp5j2 | 673.9710759 | 1.077770192 | 0.000776967 | 0.022354028 |
| Cox6b1 | 1155.557459 | 1.076069391 | 0.001570273 | 0.036577478 |
| Trf | 2687.068747 | 1.07574641 | 0.000919716 | 0.02490711 |
| Rpl4 | 2883.694752 | 1.073164979 | 0.001350173 | 0.033396742 |
| mt-Rnr1 | 61932.63189 | 1.060827982 | 0.001150499 | 0.029631271 |
| Rps9 | 1965.002266 | 1.051650812 | 0.002179002 | 0.044175425 |
| Rps10 | 3700.203249 | 1.051168452 | 0.000182443 | 0.008093437 |
| Rpl11 | 3629.485584 | 1.045954603 | 0.001917219 | 0.041740247 |
| Msn | 2102.240851 | 1.031713583 | 0.001008643 | 0.027022427 |
| Dnajc13 | 1061.122093 | 1.023554222 | 0.002172093 | 0.044175425 |
| Hint1 | 1396.020053 | 1.021263743 | 0.000154276 | 0.007170609 |
| Rps25 | 5668.244204 | 0.952581426 | 0.002005166 | 0.042645062 |
| mt-Nd2 | 13095.73537 | 0.94264793 | 0.002500295 | 0.048421411 |
| mt-Nd4 | 8805.176595 | 0.933049814 | 0.001093239 | 0.028707698 |
| Anp32b | 1816.361714 | 0.923737089 | 0.001610079 | 0.037244251 |
| Npc2 | 5059.366253 | 0.919098637 | 0.00117679 | 0.029922807 |
| Pon2 | 1056.740227 | 0.909650157 | 0.001530855 | 0.036079803 |
| Atp5a1 | 1669.93379 | 0.884729659 | 0.001508207 | 0.035884547 |
| Cyba | 2059.854378 | 0.882103987 | 0.002067335 | 0.042949843 |
| Gpx1 | 2840.266396 | 0.878808546 | 0.002171711 | 0.044175425 |
| Nop58 | 1707.572909 | 0.846900659 | 0.001843489 | 0.040577053 |
| Ctss | 78466.23865 | 0.70625493 | 0.001567971 | 0.036577478 |
| Irf8 | 3254.722937 | -0.796405654 | 0.001450976 | 0.035108 |
| Atf3 | 7617.357008 | -0.802408563 | 0.000658246 | 0.020177474 |
| Ddx5 | 6680.618519 | -0.838128272 | 0.001064455 | 0.028290156 |
| Mef2c | 2016.465876 | -0.900136607 | 0.000289438 | 0.011298248 |
| Sirpa | 5933.697522 | -0.909080694 | 0.000435755 | 0.015172488 |
| Basp1 | 2937.775829 | -0.925687242 | 0.001882876 | 0.041236008 |
| Asph | 2255.48857 | -0.930275944 | 0.000186946 | 0.008229724 |
| Glul | 5293.120468 | -0.930480662 | 7.68E-05 | 0.004147502 |
| St3gal6 | 847.2825158 | -0.950357013 | 0.002572686 | 0.049726994 |
| Rgs2 | 12378.25691 | -0.950682344 | 1.74E-05 | 0.001362745 |
| Jun | 45176.13823 | -0.954693745 | 0.000114675 | 0.005701214 |
| Ssh2 | 3997.274501 | -0.96927919 | 0.000260952 | 0.010389221 |
| Sema4c | 1128.943891 | -0.984513711 | 0.002269786 | 0.045363943 |
| H2-K1 | 6229.98568 | -1.007867463 | 0.000381766 | 0.01403027 |
| Hpgds | 1809.595919 | -1.008456173 | 0.000415793 | 0.014839369 |
| Ythdc1 | 1113.370345 | -1.034417911 | 0.002129361 | 0.043714411 |
| Egr1 | 17693.46669 | -1.055907261 | 0.002130383 | 0.043714411 |
| Zfp710 | 514.3054817 | -1.059074046 | 0.001754715 | 0.039140335 |
| Mycbp2 | 1429.831079 | -1.071552832 | 0.000919023 | 0.02490711 |
| Klf2 | 1892.523233 | -1.072966844 | 0.000157622 | 0.007258614 |
| Hspa8 | 3502.178981 | -1.07478882 | 0.000448841 | 0.015519972 |
| Cttnbp2nl | 1865.083723 | -1.106820313 | 0.00024612 | 0.009957381 |
| Ppp1r15a | 4014.377754 | -1.107308138 | 0.001287296 | 0.032240484 |
| Csnk1e | 905.9925583 | -1.116637422 | 0.00015693 | 0.007258614 |
| Tubb2a | 1395.286241 | -1.118318166 | 0.000188856 | 0.008276044 |
| Ccl4 | 7595.52891 | -1.119383104 | 0.000128289 | 0.00625363 |
| Btg2 | 4854.667109 | -1.123162426 | 5.48E-05 | 0.003140966 |
| Lhfpl2 | 1023.783408 | -1.139843924 | 0.00018304 | 0.008093437 |
| Pkp4 | 928.285288 | -1.149757162 | 0.00157945 | 0.036620529 |
| Gpr34 | 1398.593715 | -1.162563046 | 0.001425869 | 0.034811057 |
| Akna | 1723.747835 | -1.167378632 | 0.002059065 | 0.042949843 |
| Trib1 | 2630.359874 | -1.18768932 | 0.00152859 | 0.036079803 |
| Plxdc2 | 2128.366027 | -1.187881237 | 1.31E-05 | 0.001138413 |
| Kif21b | 587.4890831 | -1.218535115 | 0.001172131 | 0.029903641 |
| Rhob | 6058.266549 | -1.223156991 | 3.69E-05 | 0.002361649 |
| Zbtb11 | 385.0215595 | -1.254631344 | 0.000621853 | 0.019419311 |
| Herpud1 | 2709.915831 | -1.279342506 | 0.000577563 | 0.018498675 |
| Slco2b1 | 3688.56432 | -1.299158177 | 1.22E-07 | 2.18E-05 |
| Klf6 | 11777.55684 | -1.31600389 | 9.33E-08 | 1.79E-05 |
| Txnip | 2384.50699 | -1.332609515 | 0.002043303 | 0.042920833 |
| Cd180 | 733.2981707 | -1.33359512 | 0.000570237 | 0.01838186 |
| Sgk1 | 1429.954238 | -1.338708638 | 0.000758135 | 0.02218136 |
| Il6st | 617.2278248 | -1.343764972 | 0.00035231 | 0.013235458 |
| Ccnl2 | 288.5853189 | -1.346761532 | 0.002110878 | 0.043714411 |
| Orai1 | 1202.815517 | -1.353369505 | 0.000759134 | 0.02218136 |
| Srgap2 | 3693.966261 | -1.357834794 | 6.48E-07 | 9.52E-05 |
| Kctd12 | 6533.419255 | -1.358711331 | 4.77E-06 | 0.000512705 |
| Lpin2 | 531.936807 | -1.391439674 | 0.000141719 | 0.00680255 |
| Bbc3 | 1549.107701 | -1.394421966 | 0.000217387 | 0.009017989 |
| Fam102b | 776.4808557 | -1.396134976 | 6.91E-05 | 0.003775863 |
| Il10ra | 888.7274351 | -1.399687978 | 0.000593299 | 0.018831566 |
| Git2 | 349.9183111 | -1.416737731 | 0.002242763 | 0.045185351 |
| Abca9 | 1163.600359 | -1.435100751 | 1.23E-06 | 0.00015506 |
| Nuak1 | 809.1889493 | -1.436373137 | 0.00072204 | 0.021538351 |
| Il17ra | 2051.306792 | -1.473096795 | 1.54E-06 | 0.000192597 |
| Nsmaf | 365.4468901 | -1.475358261 | 0.000749994 | 0.022108228 |
| Ccl3 | 2828.197254 | -1.481913008 | 2.02E-05 | 0.001498902 |
| Senp2 | 1044.774959 | -1.483884246 | 0.00016147 | 0.007367919 |
| Mknk1 | 374.3296691 | -1.48887668 | 0.000779129 | 0.022354028 |
| Camsap1 | 385.1622788 | -1.507500309 | 0.001836636 | 0.040515455 |
| Traf3 | 336.990252 | -1.512425483 | 0.000555348 | 0.018122446 |
| Ccr5 | 1562.462641 | -1.516976369 | 1.61E-05 | 0.001313085 |
| Slc12a2 | 684.1550537 | -1.521275089 | 5.09E-05 | 0.002991411 |
| Itga6 | 922.7850718 | -1.538105471 | 2.56E-05 | 0.001750972 |
| Narf | 456.2649436 | -1.545474307 | 0.001709265 | 0.038491214 |
| Cd164 | 782.9387465 | -1.553855979 | 0.00019314 | 0.008355194 |
| Fbrsl1 | 459.5076826 | -1.577766696 | 0.001084673 | 0.028599305 |
| Ppcdc | 261.892533 | -1.578602554 | 0.000910019 | 0.024852337 |
| Rapgef5 | 531.5138269 | -1.58929006 | 0.000872505 | 0.02405763 |
| Mob3c | 1006.287385 | -1.625402872 | 0.001156202 | 0.029690734 |
| Gas6 | 650.2297867 | -1.634095671 | 0.00059361 | 0.018831566 |
| 4632427E13Rik | 213.0200966 | -1.66730215 | 0.001811364 | 0.04004637 |
| Tnfaip2 | 243.2865249 | -1.675786357 | 0.000331481 | 0.012595015 |
| Vps37b | 457.4948902 | -1.679767554 | 0.001053548 | 0.028150022 |
| Irs2 | 277.5163537 | -1.687730089 | 0.001400029 | 0.03437466 |
| P2ry13 | 3243.266162 | -1.752710443 | 7.72E-09 | 2.49E-06 |
| Ptger4 | 253.1245464 | -1.752924638 | 0.000846844 | 0.02367092 |
| Slamf8 | 261.6727265 | -1.822310017 | 0.000269402 | 0.010683089 |
| Slc22a17 | 173.248916 | -1.850198224 | 0.002053513 | 0.042949843 |
| Arrdc3 | 335.4112563 | -1.882829506 | 0.000288586 | 0.011298248 |
| Pak1 | 281.5013984 | -1.896012408 | 0.000688521 | 0.020786673 |
| Exoc7 | 136.0875942 | -1.898431144 | 0.001071572 | 0.028403769 |
| Prr12 | 122.1618137 | -1.935758252 | 0.001956135 | 0.042182161 |
| Spsb1 | 138.4221205 | -1.979098864 | 0.00203679 | 0.042920833 |
| Ets1 | 532.1330238 | -1.987067197 | 3.23E-06 | 0.000358859 |
| Man1c1 | 409.4572973 | -1.994318016 | 2.14E-05 | 0.001528348 |
| Prkca | 217.5350501 | -2.002189336 | 0.000637969 | 0.019737549 |
| Slc25a44 | 221.5642128 | -2.051776041 | 0.001615456 | 0.037282346 |
| Il18 | 152.8172011 | -2.054897068 | 0.000170946 | 0.007760349 |
| Plau | 303.1695251 | -2.058330361 | 7.00E-06 | 0.000672216 |
| Ppard | 156.6047334 | -2.103712269 | 0.002330672 | 0.046303003 |
| Idh2 | 297.0117744 | -2.122524436 | 0.001885806 | 0.041236008 |
| Cdc23 | 158.5617852 | -2.146128391 | 0.000553658 | 0.018122446 |
| Mgll | 251.4263844 | -2.154811256 | 5.73E-05 | 0.003211581 |
| Zfp691 | 219.5530788 | -2.165150151 | 0.000199789 | 0.008532021 |
| Nfatc2 | 147.6146632 | -2.1725986 | 0.000628329 | 0.019499661 |
| Adgrg1 | 1584.5415 | -2.173665092 | 4.58E-08 | 1.02E-05 |
| Slc1a3 | 231.2391089 | -2.174444759 | 0.00024558 | 0.009957381 |
| Csmd3 | 414.6822793 | -2.196397968 | 3.00E-05 | 0.001974486 |
| H2-Q7 | 203.9319957 | -2.201820833 | 0.000205094 | 0.008721296 |
| Lrba | 239.3908951 | -2.203870156 | 7.86E-06 | 0.00072691 |
| Col27a1 | 661.1114619 | -2.210342884 | 2.44E-12 | 1.41E-09 |
| Tlr12 | 826.9339325 | -2.217852202 | 1.25E-05 | 0.001092656 |
| Ifit2 | 193.4778065 | -2.245907081 | 0.00189326 | 0.041308607 |
| Marcksl1 | 193.0519622 | -2.262322577 | 9.28E-05 | 0.004807504 |
| Adrb1 | 152.5634157 | -2.273912845 | 0.000780702 | 0.022354028 |
| Chst11 | 157.9855593 | -2.280088047 | 0.000573578 | 0.0184301 |
| St8sia6 | 150.1259379 | -2.303390297 | 0.000293046 | 0.011394573 |
| Gbp3 | 99.11033997 | -2.306081946 | 0.000345364 | 0.013023464 |
| Zfp869 | 93.49380043 | -2.385959546 | 0.002252174 | 0.045283653 |
| Il13ra1 | 241.9443751 | -2.426269481 | 2.61E-05 | 0.001773874 |
| Mitf | 216.6553002 | -2.502047546 | 0.00074059 | 0.021912203 |
| Pex12 | 72.76434031 | -2.507455067 | 0.002034227 | 0.042920833 |
| Gbp7 | 233.6377205 | -2.537325599 | 4.55E-05 | 0.002789675 |
| Kcnj2 | 246.6581704 | -2.644844216 | 2.53E-05 | 0.001740265 |
| Fat3 | 135.3072652 | -2.675123003 | 0.000420018 | 0.014936811 |
| Myo1b | 461.7221278 | -2.706752504 | 6.42E-08 | 1.33E-05 |
| Mtss1 | 242.9002204 | -2.713024487 | 0.001441049 | 0.034952443 |
| Liph | 231.7797796 | -2.816227527 | 1.66E-05 | 0.001335649 |
| Sox4 | 438.7483177 | -2.861474535 | 6.52E-08 | 1.33E-05 |
| Cdh2 | 164.947371 | -2.864967792 | 0.001476428 | 0.035466225 |
| Ppm1l | 213.5581101 | -2.874217252 | 0.000223353 | 0.009223018 |
| Eml6 | 130.3936914 | -2.969423298 | 0.000824878 | 0.02321974 |
| Cfap74 | 187.548654 | -3.001572945 | 6.33E-06 | 0.000634671 |
| Gbp9 | 239.6736871 | -3.007755665 | 6.59E-06 | 0.000652176 |
| Ptprm | 210.4751216 | -3.012377349 | 3.88E-05 | 0.002455237 |
| Chst2 | 114.3188236 | -3.092271335 | 0.000480992 | 0.016348836 |
| Ecscr | 54.02938932 | -3.202460801 | 0.000381891 | 0.01403027 |
| Aasdh | 83.99804139 | -3.571240298 | 0.00186368 | 0.040931335 |
| Ildr2 | 214.699315 | -3.652765753 | 3.01E-06 | 0.00033809 |
| Nav3 | 617.3610858 | -3.694004294 | 1.16E-24 | 2.33E-21 |
| Chst8 | 82.37702567 | -3.718555049 | 0.002016691 | 0.042787255 |
| Arhgef17 | 91.07880294 | -3.743129829 | 0.000463893 | 0.015966158 |
| Afap1 | 57.53365652 | -3.791759268 | 0.00127117 | 0.031916589 |
| Gm10605 | 73.50698392 | -3.945856324 | 0.00062751 | 0.019499661 |
| Zdhhc14 | 103.7521213 | -3.946706797 | 8.11E-07 | 0.000109487 |
| Gpr162 | 61.93901198 | -4.111542529 | 0.000873904 | 0.02405763 |
| Pard6a | 63.71891315 | -4.116833083 | 0.001679462 | 0.038202685 |
| Fermt2 | 44.77324502 | -4.241557477 | 0.002179374 | 0.044175425 |
| Sncaip | 55.16276582 | -4.317640282 | 9.52E-05 | 0.004879346 |
| Satb1 | 50.77468252 | -4.38352493 | 2.71E-05 | 0.001825042 |
| Igf2bp2 | 33.59441753 | -4.559688094 | 0.000969702 | 0.026189827 |
| Yod1 | 74.4489508 | -4.59348548 | 0.000910233 | 0.024852337 |
| Gm5426 | 104.3949158 | -4.861932527 | 0.001958623 | 0.042182161 |
| Hpgd | 594.4859544 | -5.132765555 | 8.34E-22 | 1.19E-18 |
| Ccl9 | 114.1534213 | -5.287771181 | 1.27E-08 | 3.53E-06 |
| Gm42466 | 72.89395328 | -5.371898567 | 0.000344619 | 0.013023464 |
| Gm45540 | 73.20401075 | -5.542458346 | 4.51E-06 | 0.000489564 |
| Med24 | 49.96515856 | -5.647347291 | 0.000385242 | 0.014101542 |
| Adamts16 | 111.3680441 | -5.768853126 | 9.35E-08 | 1.79E-05 |
| Rasip1 | 46.82840386 | -5.799367845 | 0.000125878 | 0.006194355 |
| Setbp1 | 25.64521131 | -5.841899768 | 0.001345509 | 0.033363957 |
| Rnd3 | 72.4781202 | -6.020506965 | 0.000693195 | 0.020864742 |
| Bank1 | 44.17626525 | -6.429956938 | 8.46E-05 | 0.004482016 |
| Tatdn3 | 74.61145525 | -6.532665423 | 6.66E-07 | 9.58E-05 |
| Gm13498 | 44.26185831 | -6.91565582 | 0.000547643 | 0.018122446 |
| Sec16b | 54.56727046 | -6.945115626 | 0.000146756 | 0.006885109 |
| Ifit3b | 38.5845454 | -6.954013037 | 5.18E-06 | 0.000544648 |
| Cc2d2a | 29.24581818 | -7.177973996 | 0.000614182 | 0.019239866 |
| Whrn | 75.32776677 | -7.363046585 | 1.14E-08 | 3.45E-06 |
| Lmo7 | 36.03437475 | -7.857405591 | 0.001124691 | 0.02919231 |
| Shroom1 | 43.5843442 | -8.157887913 | 0.000207229 | 0.008774756 |
| Gm12331 | 51.44551327 | -8.414660856 | 0.00038916 | 0.014192989 |
| Il7r | 62.89181899 | -8.636465651 | 5.38E-07 | 8.14E-05 |
| Vasn | 17.27337845 | -9.846312136 | 0.002403608 | 0.047189115 |
| Gm37645 | 19.26170026 | -10.00409002 | 0.002372574 | 0.046763582 |
| Gm3739 | 20.58436407 | -10.10055592 | 0.000914371 | 0.02489729 |
| Slamf7 | 23.61202406 | -10.29830905 | 0.002441937 | 0.047660703 |
| Gdpd1 | 24.34859828 | -10.34414202 | 0.000649722 | 0.019977463 |
| 4930432K21Rik | 24.96498259 | -10.37907676 | 0.001941759 | 0.0420911 |
| Klhl3 | 25.95557733 | -10.43472892 | 0.002353008 | 0.046561608 |
| Rxrg | 27.64079665 | -10.52779024 | 0.001364259 | 0.033661816 |
| Myl2 | 28.20735816 | -10.55499722 | 0.001541226 | 0.036153694 |
| Dnah10 | 29.11660664 | -10.60057955 | 0.000850618 | 0.02367751 |
| RP23-187G5.2 | 29.77619217 | -10.63294143 | 0.001530246 | 0.036079803 |
| Oasl1 | 30.48054302 | -10.66670347 | 0.001517768 | 0.036026264 |
| Gm42572 | 41.54301847 | -11.11363054 | 0.000670534 | 0.020379787 |
| Farp1 | 50.86876563 | -11.4056485 | 0.000143675 | 0.006804485 |
| Kcnma1 | 53.02313757 | -11.46528107 | 9.95E-05 | 0.005021272 |
| Tnfrsf17 | 79.19629552 | -12.04445562 | 1.72E-05 | 0.001362745 |
